# Expression profile analysis reveals that *Aspergillus fumigatus* but not *Aspergillus niger* makes type II epithelial lung cells less immunological alert

**DOI:** 10.1186/s12864-018-4895-3

**Published:** 2018-07-13

**Authors:** Natalia Escobar, Ivan D. Valdes, Esther M. Keizer, Soledad R. Ordonez, Robin A. Ohm, Han A. B. Wösten, Hans de Cock

**Affiliations:** 1 Microbiology & Institute of Biomembranes, Department of Biology, Utrecht University, Padualaan 8, 3584 CH Utrecht, The Netherlands; 2 Department of Infectious Diseases and Immunology, Division Molecular Host Defence, Utrecht University, Yalelaan 1, 3584CL Utrecht, The Netherlands

**Keywords:** Epithelial cells, Aspergillosis, *A. fumigatus*, *A. niger*, Host-microbe interaction

## Abstract

**Background:**

*Aspergillus fumigatus* is the main causative agent of aspergillosis. Infections rarely occur in immunocompetent individuals, indicating efficient clearance of conidia by pulmonary defense mechanisms. Other aspergilli like *Aspergillus niger* also cause infections but to a much lesser extent. Our previous studies showed that *A. fumigatus* and *A. niger* have different behavior in the presence of type II alveolar A549 epithelial cells. *A. fumigatus* conidia are more efficiently internalized by these cells and germination is delayed when compared to *A. niger*. In addition, hyphae that have escaped the epithelial cells grow parallel to the epithelium, while *A. niger* grows away from this cell layer*.*

**Results:**

Here it is shown that global gene expression of *A. fumigatus* and *A. niger* is markedly different upon contact with A549 cells. A total of 545 and 473 genes of *A. fumigatus* and *A. niger*, respectively, were differentially expressed when compared to growth in the absence of A549 cells. Notably, only 53 genes (approximately 10%) were shared in these gene sets. The different response was also illustrated by the fact that only 4 out of 75 GO terms were shared that were enriched in the differentially expressed gene sets. The orthologues of *A. fumigatus* genes involved in hypoxia regulation and heat shock were also up-regulated in *A. niger,* whereas thioredoxin reductase and allergen genes were found up-regulated in *A. fumigatus* but down-regulated in *A. niger*. Infection with *A. fumigatus* resulted in only 62 up and 47 down-regulated genes in A549. These numbers were 17 and 34 in the case of *A. niger.* GO terms related with immune response were down-regulated upon exposure to *A. fumigatus* but not in the case of *A. niger.* This indicates that *A. fumigatus* reprograms A549 to be less immunologically alert.

**Conclusions:**

Our dual transcriptomic analysis supports earlier observations of a marked difference in life style between *A. fumigatus* and *A. niger* when grown in the presence of type II epithelial cells. The results indicate important differences in gene expression, amongst others down regulation of immune response genes in lung epithelial cells by *A. fumigatus* but not by *A niger.*

**Electronic supplementary material:**

The online version of this article (10.1186/s12864-018-4895-3) contains supplementary material, which is available to authorized users.

## Background

*Aspergillus fumigatus* is an opportunistic pathogen that is distributed globally and found in a large variety of substrates such as soil, organic matter, and water bodies [1]. This saprophytic fungus mainly reproduces asexually by producing large amounts of 2–3 μm wide conidia that are dispersed to enable colonization of new substrates [2]. Conidia are effectively dispersed by air flows. The fact that outdoor air contains on average more than 10 *A. fumigatus* conidia m^− 3^ implies that humans inhale several hundred of these conidia each day [3, 4]. Immunocompromised individuals, like patients suffering from neutropenia due to chemotherapy or genetic disorders, cystic fibrosis, AIDS, or hematological malignancy are at high risk of acquiring invasive aspergillosis [5]. *A. fumigatus* is recognized as the causative agent in 90% of the cases of aspergillosis, indicating that this fungus has acquired a specific life style that favors colonization of humans and allows establishment of serious infections [6]. Other *Aspergillus* species (e.g. *Aspergillus flavus, Aspergillus niger, Aspergillus tubingensis*) also cause infections but to a much lower extent [7–9]. Mortality rates of intensive care unit patients with aspergillosis range between 30 and 90% depending on the immune state of the host and the stage of the infection [10].

Inhaled conidia that are not cleared in the upper respiratory area will reach the alveoli where they encounter type I and II alveolar epithelial cells. Type I cells which represent 95% of the alveolar surface are in charge of gas exchange between the alveolus and the pulmonary capillaries [11]. On the other hand, type II cells play multiple roles in the lung, amongst others they secrete pulmonary surfactant and mediate lung immunity [12–15]. An encounter with lung epithelial cells may result in clearance of the *A. fumigatus* conidia or, with low incidence, in initiation of infection. Adhesion, internalization, and germination are the 3 main steps of initiation of an *Aspergillus* infection. These steps comprise transitions from dormant conidia into swollen conidia and subsequent formation of hyphae. These morphotype transitions are expected to be accompanied by specific genetic responses of both the host and the pathogen. Previous transcriptomics studies, using fluorescence activated sorted epithelial cells containing conidia and microarrays, revealed that 889 genes of immortal bronchial epithelial 16HBE14o- cells were differentially expressed after 6 h incubation with conidia of *A. fumigatus.* Genes associated with repair and inflammatory processes such as matrix metalloproteinases, chemokines, and glutathione S-transferase were found up-regulated [16]. In another dual transcriptomic study using microarrays and 16HBE14o- cells in a similar exposure set up, a total of 255 epithelial genes were differentially expressed as compared to non-infected cells. Among the most highly enriched functional gene groups were terms involved in innate immune response, defense response, and the inflammatory response. On the other hand, 150 *A. fumigatus* genes were up-regulated, while 33 were down-regulated [17]. Amongst others, genes involved in vacuolar acidification and metallopeptidase activity were found to be differentially expressed. These studies represented an early step in the infection process. Here, gene expression was studied at a later stage of initiation of infection that included hyphal growth (i.e. after 12 h) using both *A. fumigatus* and *A. niger.* These fungi show striking differences in germination and outgrowth in the presence of type II alveolar A549 epithelial cells. *A. fumigatus* conidia are more efficiently internalized and show delayed germination when compared to *A. niger*. In addition, hyphae that have escaped from the epithelial cells grow parallel to the epithelium, while *A. niger* hyphae grow perpendicular to this cell layer [18]*.* Here it is shown that global gene expression of *A. fumigatus* and *A. niger* is markedly different upon a 12 h contact with epithelial cells. From a total of 545 (*A. fumigatus)* and 473 (*A. niger)* differentially expressed genes, only 53 genes were shared by both species after co-cultivation with A549 cells. Moreover, A549 cells responded differently to the two fungi illustrated by the down regulation of immune response genes in the case of *A. fumigatus* but not in the case of *A. niger* exposure. Together, this supports the view that *A. fumigatus* and *A. niger* have a different life style when exposed to epithelial cells.

## Methods

### Strains and growth conditions

*A. fumigatus* Af293 [19] and *A. niger* N402 [20] were grown on potato dextrose agar (PDA) (Becton, Dickinson and company, Le-Pont-De-Claix, France) for 3 days at 37 °C. Conidia were harvested with 0.85% (*w*/*v*) NaCl and filtered through 3 layers of Miracloth (Merck Millipore Corporation, Darmstadt, Germany) to remove pieces of mycelium. Suspensions were adjusted to 10^8^ conidia ml^− 1^ after counting with a Bürker chamber and this was checked by plating on PDA.

### Cell cultures and fungal co-cultivation

The human lung carcinoma epithelial type II cell line A549 (ATCC, CCL-185) was maintained by serial passages as described [18]. A549 cells were seeded at a concentration of 2 × 10^5^ cells mL^− 1^ in 6-well plates (Corning® Costar ®, New York, USA) and cultured in 2 mL Dulbecco’s Modified Eagle’s Medium (DMEM) with 10% FCS at 37 °C and 5% CO_2._ After a confluent monolayer was formed, cells were challenged for 2 h with 2 × 10^5^ to 10^6^ fungal conidia well^− 1^ (MOI of approximately 0.1 to 1 dependent of the experimental set up). Unbound conidia were removed carefully by washing 3 times with DMEM (pre-warmed to 37 °C), after which 2 mL DMEM with 10% FCS was added to the well. Growth was prolonged for 10 h. A549 and fungal conidia grown individually under the same culture conditions served as controls. In the latter case, *A. fumigatus* was grown until hyphae reached a length similar to those in the presence of A549 cells. Cells were harvested by scraping in the presence of lysis/binding buffer (4.5 M guanidine-HCl, 100 mM sodium phosphate pH 6.6) from the High Pure RNA tissue kit (Roche Diagnostics GmbH, Mannheim, Germany), frozen in liquid nitrogen, and stored at − 80 °C. For the 8 h co-cultivation of the fungus with the A549 cells unbound conidia were removed by carefully washing after 8 h of incubation, before the start of the RNA isolation, as described by Chen et al. (2015) [21]. Conidia harvested with 0.85% (*w*/*v*) NaCl with 0.1% Tween 20 or Tween 80 were washed twice with 0.85% (w/v) NaCl and filtered through 3 layers of Miracloth (Merck Millipore Corporation, Darmstadt, Germany). Suspensions were adjusted to 10^8^ conidia mL^− 1^ after counting with a Bürker chamber and determining the CFU mL^− 1^. A549 cells were incubated for 12 h with different concentrations Tween-20 and Tween-80 in culture medium before RNA isolation.

### RNA extraction and RNA-sequencing

Cells were homogenized in an Eppendorf tube with a Tissuelyser for 5 min at 20 Hz min^− 1^ (Qiagen, Venlo, The Netherlands) using 2 metal beads (4.76 mm in diameter). The homogenate was incubated for 5 min at RT after adding 1 mL of TRIzol (Thermo Fisher Scientific, Waltham, MA, USA), followed by addition of 200 μL chloroform, and an incubation of 3 min. Samples were centrifuged for 10 min at 10.000 g and 4 °C and the aqueous phase was transferred to a new Eppendorf tube. RNA was purified with the RNeasy NucleoSpin® RNA kit (Macherey-Nagel GmbH & Co. KG, Düren, Germany) with some modifications. Briefly, 350 μL of 75% ethanol was added to the aqueous phase and mixed by vortexing. Sample was loaded to a NucleoSpin® RNA column and centrifuged for 30 s at 11.000 g. Then, 350 μL of membrane desalting buffer (MDB) (chaotropic salt, EtOH 5–15%, and others) was added and centrifuged for 1 min at 11.000 g. After centrifugation, 95 μL of DNase reaction mixture (rDNase, RNase-free) was loaded onto the center of the column and incubated for 15 min at RT. 200 μL of wash buffer RAW2 (Guanidinium thiocyanate 24–36%, EtOH 20–35%, and others) was added and the column was centrifuged for 30 s at 11.000 g. A second washing step was performed with 600 μL wash buffer RA3 (EtOH 76% and others). A final washing step was performed with 250 μL RAW3 buffer and centrifugation for 2 min at 11.000 g. RNA was eluted with 40 μL RNase free H_2_O by centrifugation for 1 min at 11.000 g. The flow through was collected and passed again through the column to increase RNA concentration. RNA concentration and purity was determined with the NanoDrop™ ND1000 spectrophotometer (ThermoFischer Scientific).

### RNA sequencing and transcriptome analysis

RNA sequencing was performed at ServiceXS (Leiden, The Netherlands) using Illumina NextSeq 500 according to manufacturer protocols. The NEBNext ultra Directional RNA library Prep kit for Illumina was used for cDNA synthesis. Briefly, the poly-A containing mRNA was isolated from 10 μg total RNA using poly-dT oligo magnetic beads. After fragmentation of the mRNA, cDNA synthesis was performed. Adapters were ligated to the cDNA and amplified by PCR to create the final cDNA library. Quality and yield was measured with the Fragment Analyzer (ThermoFischer Scientific). Size of the cDNA fragments was between 300 and 500, as expected. A concentration of 1.6 pM of DNA was used for sequencing. RNA-seq was performed on RNA isolated form two independent experiments for each condition. RNA sequencing covered a depth of 10, 20, and 150 Mb in the case of the fungal samples (control), A549 samples (control), and mixed samples (co-cultivation), respectively.

Quality control of the reads (see for details Additional file 1: Table S7) was checked using fastQC version 0.11.5 (http://www.bioinformatics.babraham.ac.uk/projects/fastqc/). Reads were cleaned and trimmed using Fastx-Toolkit (http://hannonlab.cshl.edu/fastx_toolkit/). Alignment of the reads was performed with the package TopHat version 2.1.1 [20] using default parameters. The *A. fumigatus* 293 [19], *A. niger* ATCC 1051 v4.0 [22] and *Homo sapiens* vGRCh38.p5 (ftp://ftp.ensembl.org/pub/release-75//fasta/homo_sapiens/dna/) genomes were used as reference. Abundance estimation and analysis of differential expression was performed using Cufflinks 2.2.1 and Cuffdiff 2.2.1 version [21, 22] using default parameters and the output metrics from the CuffLinks Pipeline are described in Additional file 2: Figure S1. Genes were considered differentially expressed if they had a fold change of at least 2 and a minimum of 4 FPKM in at least one condition [23]. Custom scripts were developed in Python and R to analyze over- and under-representation of functional annotation terms (GO, KEGG, Secretion signals, MEROPS, Secondary metabolism) in sets of differentially regulated genes using the Fisher Exact test. The Benjamini-Hochberg correction was used to correct for multiple testing using a *p*-value of < 0.05. Gene Ontology (GO) enrichment of infected A549 cells was performed using BiNGO 3.0.3 (Plug-in of Cytoscape 3) (http://www.psb.ugent.be/cbd/papers/BiNGO/), PANTHER (http://pantherdb.org/) and REACTOME (https://reactome.org/). Lists of GO terms were cleaned from GO redundancies running REViGO (http://revigo.irb.hr/) software. Bidirectional protein blast analysis was performed using NCBI blast tool (e-value ≤10^− 5^)(http://blast.ncbi.nlm.nih.gov/Blast.cgi) to determine the orthologous genes between *A. fumigatus* Af293 and *A. niger* ATCC1015. RNA sequencing data were deposit at sequence read archive (SRA) of NCBI under accession number SRP132199.

### Validation of RNA-Seq analysis results by RT-qPCR

RNA from the infected and uninfected A549 cells was isolated with the High Pure RNA Isolation kit (Roche, Cat.No.118286655001), following manufacturer’s protocol. For quantitative PCR (RT-qPCR) analysis cDNA was synthesized using the QuantiTech Reverse Transcription Kit (Qiagen, Cat.No.205313), following manufacturer’s protocol. RT-qPCR was preformed using ABI Prism 7900HT SDS and SYBR Green chemistry (Applied Biosystems). Primers used for qPCR analysis can be found in Table 1. For the data analysis the Pfaffl method was used with the program REST2009 [24]. PO, TBP were reference genes for A549 cells, genes *tef1*, *gpdA* for *A. fumigatus* and 18S rRNA gene and the *tub2* gene for *A niger*.

**Table 1:** Primers used for RT-qPCR

Gene	Primer Sequence (From 5′ to 3′)	Reference
PO	F: GGCGACCTGGAAGTCCAACT R: CCATCAGCACCACAGCCTTC	Bellanger et al., 2009 [60]
TBP	F: GCACAGGAGCCAAGAGTGAA R: CACATCACAGCTCCCCACCA	Bellanger et al., 2009 [60]
TMEM160	F: CCTGGCTCCTCCGAAAAG R: ATGAAGGAGATGACCCCGAT	
EGR1	F: AGCCCTACGAGCACCTGAC R: GTTTGGCTGGGGTAACTGGT	
TNFAIP6	F: GGGAAGACACTCAAGGATGG R: GCTTGTATTTGCCAGACCGT	
FSIP1	F: AGCAACGCGATCTGCATAAT R: TTCAGAGAGACGTGATGTGGA	
IL8	F: CACCGGAAGGAACCATCTCACTGT R: TCCTTGGCAAAACTGCACCTTCA	Bellanger et al., 2009 [60]
TEF1	F: TTCCAAGCCCATGTGTGTCGAG R: ACGGACTTGACGACACCAACAG	
*gpdA*	F: TCTCCGTTGTCGACCTCACTTG R: TCCTCAGTGTAGCCGAGGATGTTC	
*sodB*	F: TGAACGACAAAGCCTCGTATGCC R: ATTCTCCGCTTCAGCCCAGTTG	
*dprB*	F: ACAGCTCCAACATCGCCAATAAAG R: TGTGGGCATGGTTATCTCTATCGC	
18S	F: GCTCGGCACCTTACGAGAAATC R: TTCAGCCTTGCGACCATACTCC	
*tub2*	F: ATGATGGCTGCCTCTGACTTC R: TTCTTGCTCTGGATGTTGCG	
An903	F: GTGTCTACGCTGGCTATACGAACC R: GGACACCTGTCTGCATCAAAGC	
An736	F: ATTGAGGGATGGCACACTGAGG R: TCGCATACTTTCAATGCCGCTTC	
An693	F: AATGCCTTCTGCGAGTGGAGTC R: CAGCGAAGAAACCACAAACACG	

## Results

### Differential gene expression of *A. fumigatus* and *A. niger* during co-cultivation with A549 cells

**Fig. 1:**
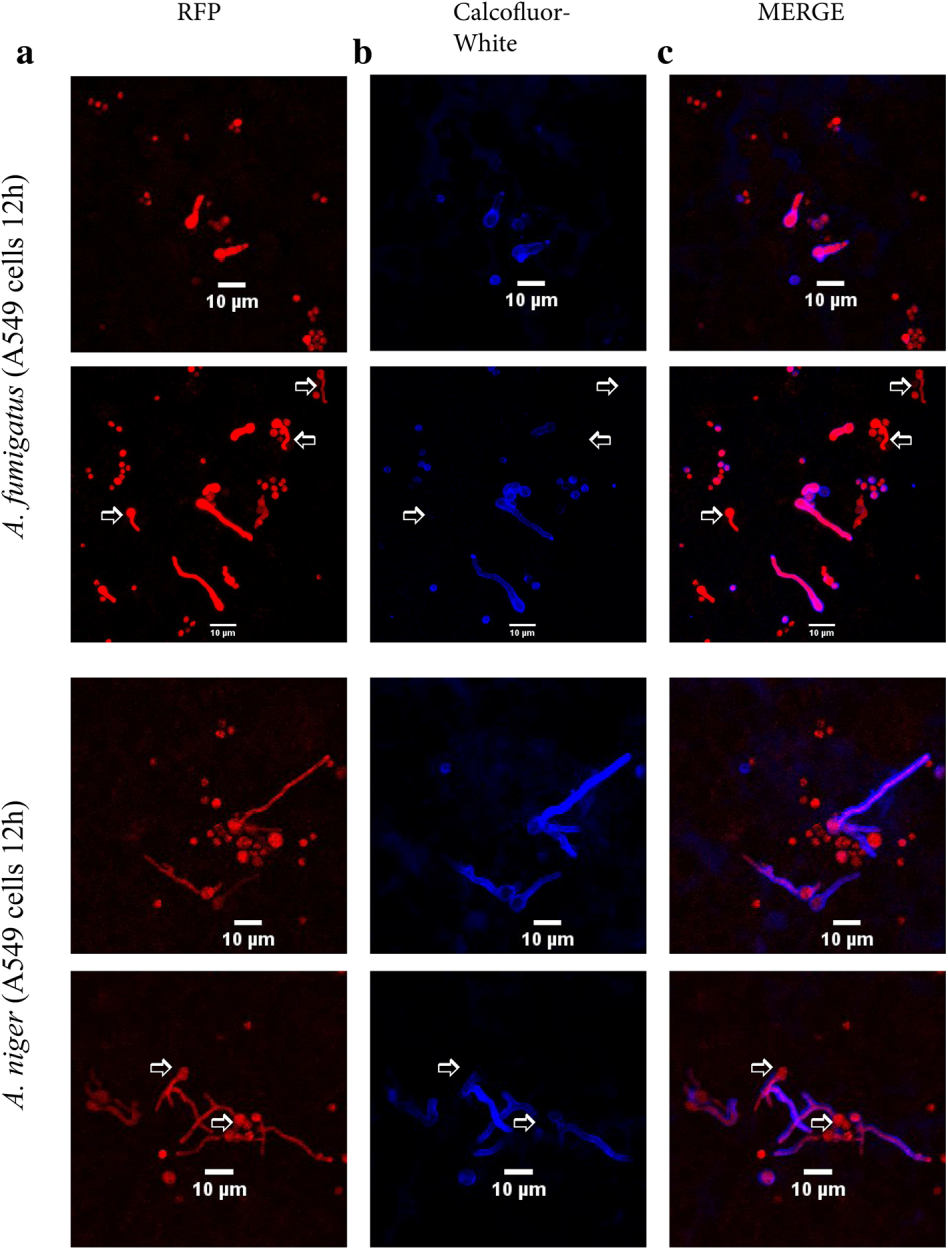
Existence of different morphotypes of *A. fumigatus* and *A. niger* when co-incubated with A549 cells after 12 h. **a** *A. fumigatus* and *A. niger* expressing red fluorescent protein (RFP), (**b**) external conidia and hyphae stained with Calcofluor-white (blue) and (**c**) overlay of the RPF and Calcoflour white images of (**a**) and (**b**) (pink colored indicates that these fungal structures are outside). Internal germinating conidia with germ tubes/hyphal structures are indicated with an arrow. Note that we selected a specific set of images here to show the presence of these different morphotypes

As previously described [18], we observed that a large proportion of conidia of *A. fumigatus* and *A. niger* were internalized and part of them had germinated 12 h after addition to A549 cells. Different fungal morphotypes were detected either inside or outside these A549 cells ranging from dormant conidia, swollen conidia as well as germ tubes and longer hyphal forms (Fig. 1). The majority of the hyphae grew outside the A549 cells, however small internal hyphae were also observed. Some of the external hyphae might also have escaped the epithelial cells and could be detected with Calcofluor-white. We addressed here differential gene expression of both fungi as well as A549 cells after a 12 h co-incubation using RNA-seq analysis. *A. fumigatus* and *A. niger* have a reduced growth rate when co-cultured with epithelial cells. Therefore, hyphae of the control condition (i.e. in the absence of A549) were grown for 10 and 9 h in the case of *A. fumigatus* and *A. niger,* respectively, to reach a length similar to that observed in the presence of epithelial cells. RNA of *A. fumigatus* and *A. niger* comprised 0.6 and 0.65% of the total number of reads after quality control (Table 2 and Additional file 1: Table S7). Thus, read number was 24 and 11 fold lower when compared to the control where the aspergilli were grown in the absence of A549.

**Table 2:** Overview of quality of the RNAseq reads and the number of reads aligning to the human and the *A. fumigatus* and *A. niger* genomes

	Total read number	Read number after cleaning	% reads aligned to A549	% reads aligned to *A. fumigatus*	% reads aligned to *A. niger*
A549	2.41*10^7^	2.41*10^7^	95.35		
*A. fumigatus*	1.32*10^7^	1.32*10^7^		96.1	
*A. niger*	3. 19*10^7^	3. 19*10^7^			95.65
A549 + *A. fumigatus*	1.93*10^8^	5.26*10^7^	94.8	0.6	
A549 + *A. niger*	1.98*10^8^	5.29*10^7^	94.75		0.65

A total number of 9840 and 11,910 genes are present in the genomes of *A. fumigatus* and *A. niger*, respectively [19, 22]. Blast analyses revealed that 62% of the genes have a bidirectional hit at protein level (e-value ≤10^− 5^). Of the *A. fumigatus* and *A. niger* genes, 9065 and 9768 were expressed in the absence of A549 cells, while only 6923 and 6448 were expressed in the presence of the epithelial cells (when > 0 FPKM was used). This huge apparent drop in expression is likely due to the 24 and 11-fold lower fungal read numbers in the co-culture. When ≥4 FPKM was used to define an expressed gene, 7059 and 6375 *A. fumigatus* genes were expressed in the absence and presence of A549. These numbers were 5939 and 5712 for *A. niger,* respectively.

**Fig. 2:**
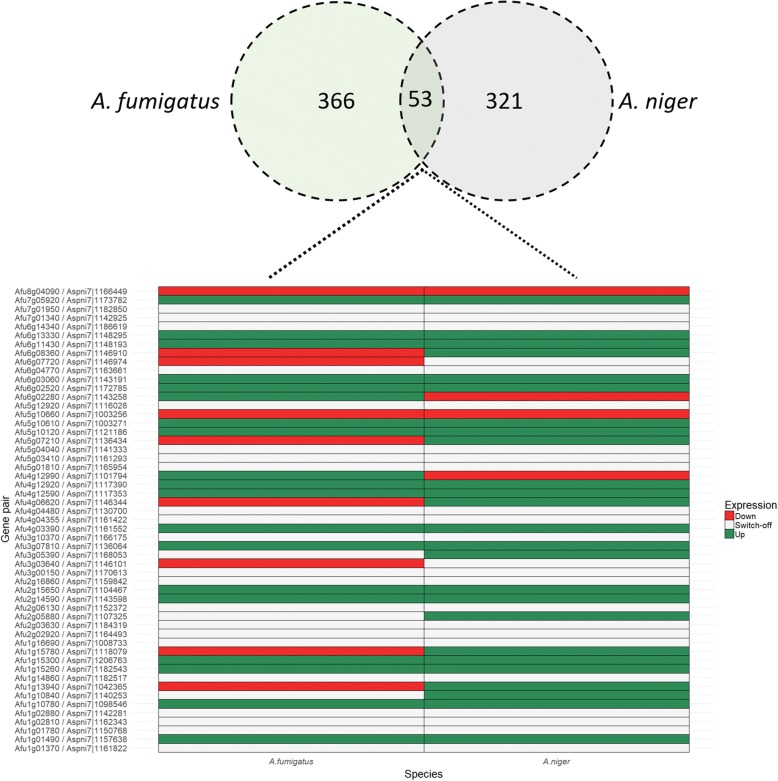
Venn diagram and heatmap of differentially expressed orthologous genes between *A. fumigatus* and *A. niger* expressed during 12 h co-incubation with A549 cells

GO terms representing biological process, cellular component, and molecular function have been annotated to 54.5 and 53% of the genes in the *A. fumigatus* and *A. niger* genomes, respectively [19, 22]. A total of 545 *A. fumigatus* genes were differentially expressed (fold change ≥2, expression ≥4 FPKM and q-value < 0.05), of which 119 were up-regulated when compared to the control condition and 426 were down-regulated (Additional file 3: Table S1). In the case of *A. niger*, 203 and 270 genes were up- and down-regulated, respectively (Additional file 4: Table S2). Bidirectional blast analysis showed that 374 out of the 473 differentially expressed genes of *A. niger* have an orthologue (e value < 10^− 5^) in the genome of *A. fumigatus.* Conversely, 419 out of the 545 differentially expressed *A. fumigatus* genes have an orthologue in the *A. niger* genome. Notably, 53 genes were shared between the set of differentially expressed genes of the two fungi (Table 3 and Fig. 2).

**Table 3:** Orthologous *A. fumigatus* and *A. niger* genes shared in the sets of differentially expressed genes when grown in the presence of A549

*A. fumigatus genes*	Fold change	Up/down regulation	*A. niger genes*	Fold change	Up/down regulation
Afu1g01490	5.05	Up	Aspni7|1,157,638	349.48	Up
Afu1g10780 (sdh1)	3.87	Up	Aspni7|1,098,546	8.69	Up
Afu1g15260	6.04	Up	Aspni7|1,182,543	14.4	Up
Afu1g15300	3.02	Up	Aspni7|1,206,763	87.12	Up
Afu2g14590	7.36	Up	Aspni7|1,143,598	3.65	Up
Afu2g15650	23.66	Up	Aspni7|1,104,467	21.14	Up
Afu3g07810	4.36	Up	Aspni7|1,136,064	3.23	Up
Afu4g03390	6.27	Up	Aspni7|1,161,552	14.33	Up
Afu4g12590	15.18	Up	Aspni7|1,117,353	48.25	Up
Afu4g12920	2.43	Up	Aspni7|1,117,390	3.52	Up
Afu4g12990 (trr1)	4.47	Up	Aspni7|1,101,794	7.80	Down
Afu5g10120	3.76	Up	Aspni7|1,121,186	30.54	Up
Afu5g10610 (rip1)	3.26	Up	Aspni7|1,003,271	3.07	Up
Afu6g02280 (aspf3)	2.41	Up	Aspni7|1,143,258	2.97	Down
Afu6g02520	2.01	Up	Aspni7|1,172,785	3.90	Up
Afu6g03060	18.45	Up	Aspni7|1,143,191	14.30	Up
Afu6g11430 (aldA)	3.61	Up	Aspni7|1,148,193	3.09	Up
Afu6g13330	7.53	Up	Aspni7|1,148,295	20.67	Up
Afu7g05920	2.03	Up	Aspni7|1,173,782	5.76	Up
Afu1g01370	Switched off	Down	Aspni7|1,161,822	Switched off	Down
Afu1g01780	Switched off	Down	Aspni7|1,150,768	Switched off	Down
Afu1g02810	Switched off	Down	Aspni7|1,162,343	Switched off	Down
Afu1g02880	Switched off	Down	Aspni7|1,142,281	Switched off	Down
Afu1g10840	Switched off	Down	Aspni7|1,140,253	39.58	Up
Afu1g13940 (sun2)	2.34	Down	Aspni7|1,042,365	29.00	Up
Afu1g14860	Switched off	Down	Aspni7|1,182,517	Switched off	Down
Afu1g15780 (leu2A)	2.60	Down	Aspni7|1,118,079	4.68	Up
Afu1g16690	Switched off	Down	Aspni7|1,008,733	Switched off	Down
Afu2g02920	Switched off	Down	Aspni7|1,164,493	Switched off	Down
Afu2g03630	Switched off	Down	Aspni7|1,184,319	Switched off	Down
Afu2g05880 (meaA)	Switched off	Down	Aspni7|1,107,325	5.16	Up
Afu2g06130	Switched off	Down	Aspni7|1,152,372	Switched off	Down
Afu2g16860	Switched off	Down	Aspni7|1,159,842	Switched off	Down
Afu3g00150	Switched off	Down	Aspni7|1,170,613	Switched off	Down
Afu3g03640 (mirB)	58.06	Down	Aspni7|1,146,101	Switched off	Down
Afu3g05390	Switched off	Down	Aspni7|1,168,053	24.65	Up
Afu3g10370	Switched off	Down	Aspni7|1,166,175	Switched off	Down
Afu4g04355	Switched off	Down	Aspni7|1,161,422	Switched off	Down
Afu4g04480	Switched off	Down	Aspni7|1,130,700	Switched off	Down
Afu4g06620	5.78	Down	Aspni7|1,146,344	6.59	Up
Afu5g01810	Switched off	Down	Aspni7|1,165,954	Switched off	Down
Afu5g03410	Switched off	Down	Aspni7|1,161,293	Switched off	Down
Afu5g04040	Switched off	Down	Aspni7|1,141,333	Switched off	Down
Afu5g07210 (met2)	9.59	Down	Aspni7|1,136,434	8.14	Up
Afu5g10660	2.93	Down	Aspni7|1,003,256	18.19	Down
Afu5g12920	Switched off	Down	Aspni7|1,116,028	Switched off	Down
Afu6g04770	Switched off	Down	Aspni7|1,163,661	Switched off	Down
Afu6g07720 (acuF)	3.56	Down	Aspni7|1,146,974	Switched off	Down
Afu6g08360	3.24	Down	Aspni7|1,146,910	7.62	Up
Afu6g14340	Switched off	Down	Aspni7|1,186,619	Switched off	Down
Afu7g01340	Switched off	Down	Aspni7|1,142,925	Switched off	Down
Afu7g01950	Switched off	Down	Aspni7|1,182,850	Switched off	Down
Afu8g04090(codA)	2.58	Down	Aspni7|1,166,449	5.90	Down

*A. fumigatus* genes and *A. niger* orthologues previously described to be induced during hypoxia (*sdh1, rip1, aldA*), to be part of the citric acid cycle (*sdh1*) and induced by heat shock (Afu4g12920) were found up-regulated in both fungi*. G*enes *trr1* (Putative thioredoxin reductase) and *aspf3* (Allergen Asp f 3; peroxiredoxin family reductase) were found up-regulated in *A. fumigatus* but their *A. niger* orthologues were down-regulated. *A. fumigatus* genes with predicted zinc ion binding activity (Afu1g01780, Afu2g06130), siderophore iron transporter (*mirB* and Afu5g12920), phosphoenolpyruvate carboxykinase (*acuF*), choline oxidase (*codA*), and major facilitator superfamily (MFS) multidrug transporter (Afu2g16860) were found down-regulated in both species. Eight genes found down-regulated in *A. fumigatus* showed orthologues up-regulated in *A. niger,* including the genes encoding an adhesin-like protein structure (*sun2*), a protein involved in leucine biosynthesis (*leu2A*), an ammonium transporter (*meaA*), and a homoserine O-acetyltransferase (*met2*).

**Fig. 3:**
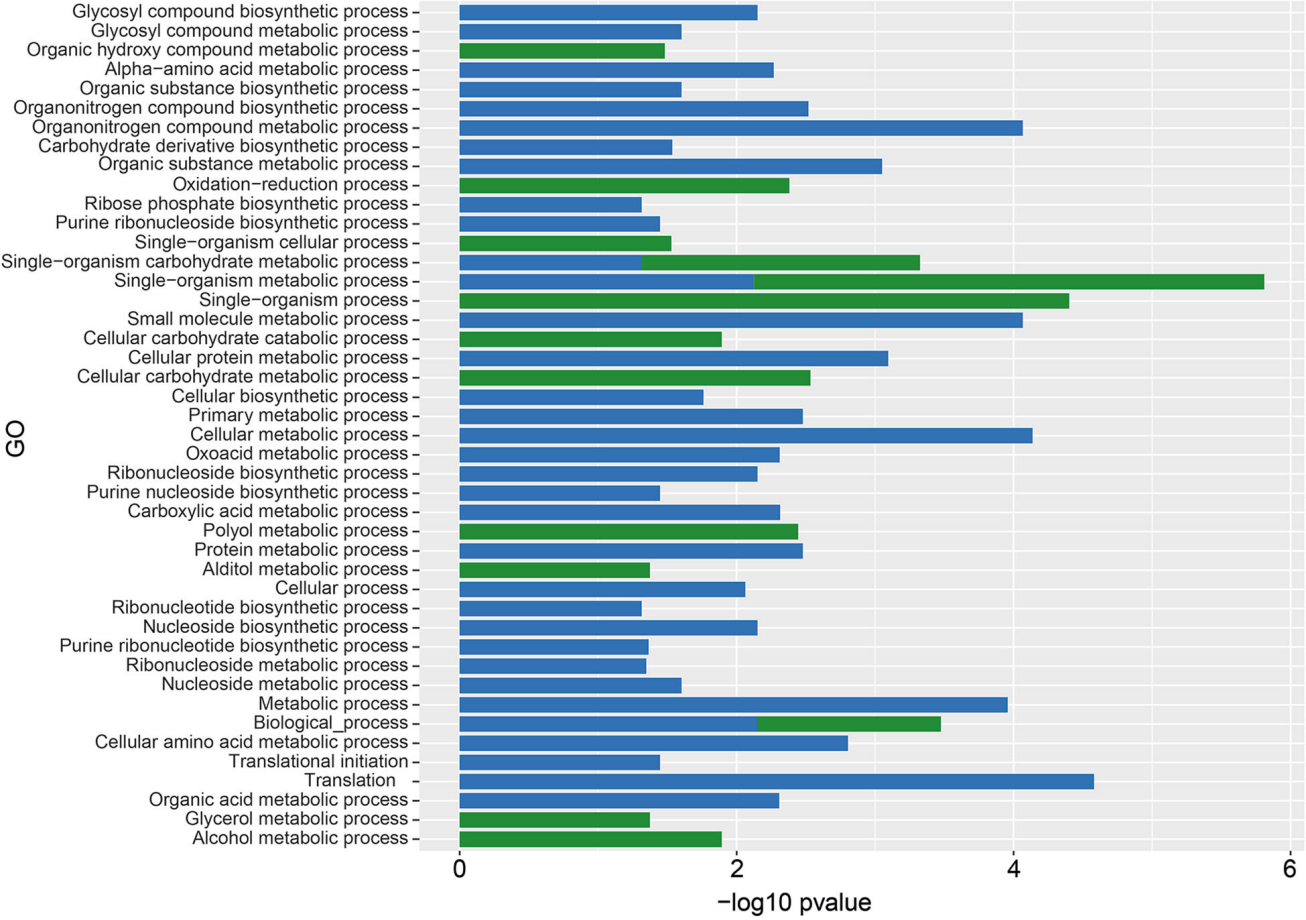
GO term enrichment analysis of biological process. *A. fumigatus* up-regulated (green) and *A. niger* up- regulated (blue) genes after 12 h of incubation in the presence of A549 epithelial cells

**Fig. 4:**
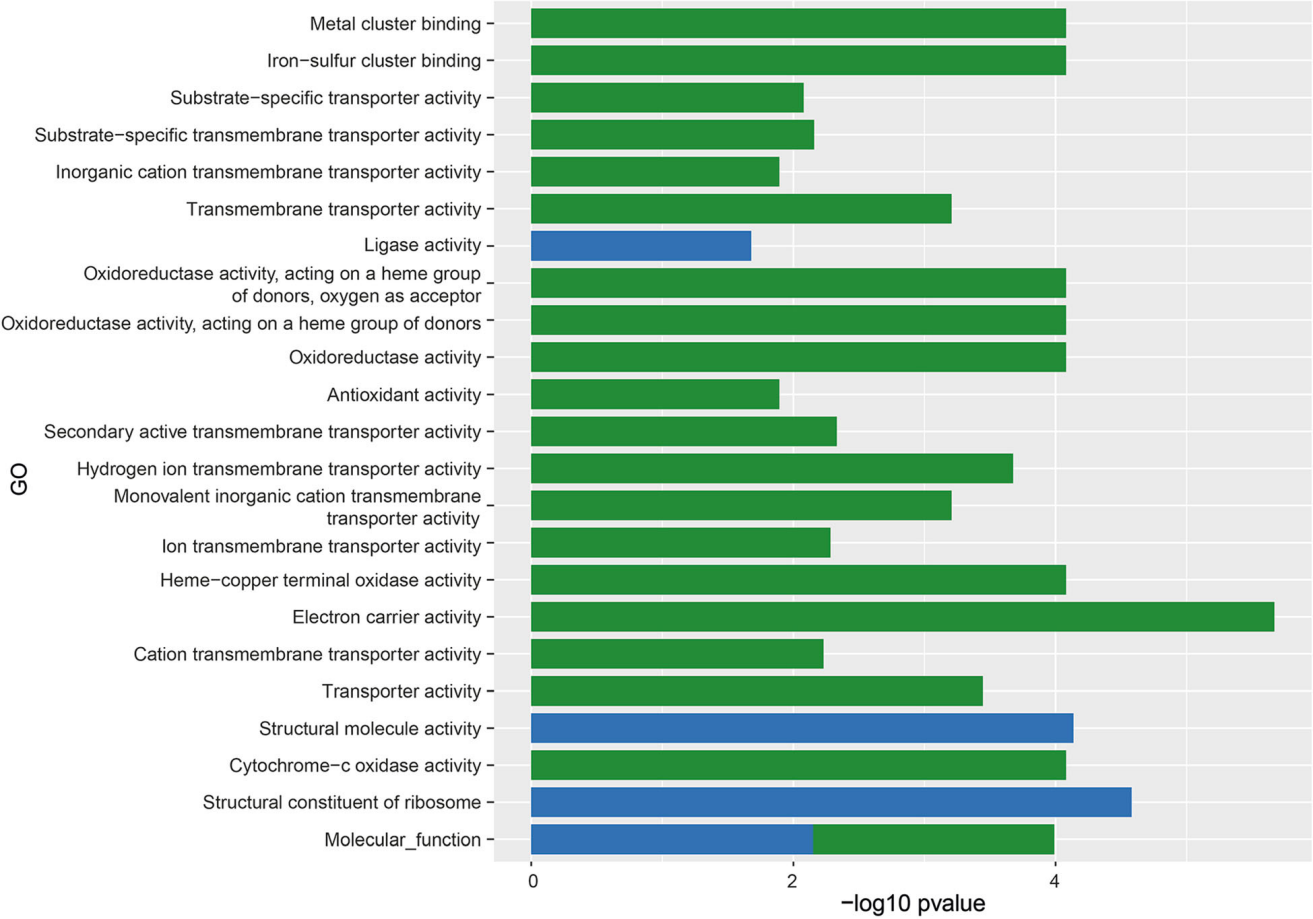
GO term enrichment analysis of molecular function. *A. fumigatus* up-regulated (green) and *A. niger* up- regulated (blue) genes after 12 h of incubation in the presence of A549 epithelial cells

**Fig. 5:**
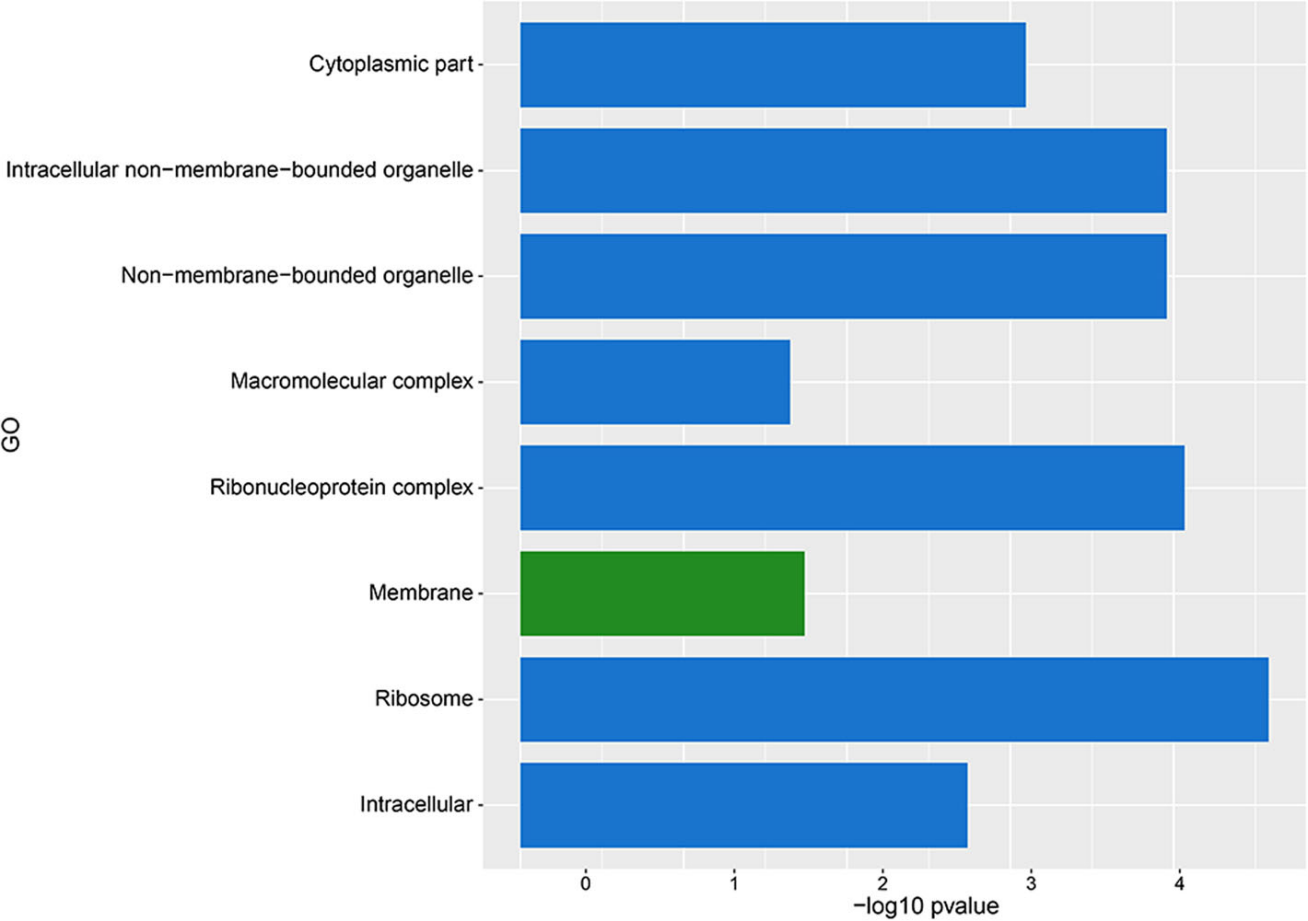
GO term enrichment analysis of cellular component. *A. fumigatus* up-regulated (green) and *A. niger* up- regulated (blue) genes after 12 h of incubation in the presence of A549 epithelial cells

Out of the 119 up-regulated and 426 down-regulated *A. fumigatus* genes 83 and 92, respectively, were classified in at least one GO term. Up-regulated genes were mainly enriched for GO terms associated with biological processes, like metabolism of organic hydroxy compounds, polyol, alditol, and alcohol (Fig. 3; Additional file 3: Table S1). GO terms associated with molecular function such as terms related to transport and the terms iron-sulfur and metal ion clustering binding, electron carrier activity, cytochrome-c oxidase activity and, heme-copper terminal oxidase activity were also over-represented in the up-regulated genes (Fig. 4). GO analysis also showed that up-regulated genes were enriched in the cellular component GO term membrane (Fig. 5).

Eight differentially expressed *A. fumigatus* genes were found switched off (i.e. 0 FPKM) in the absence of A549 but expressed (i.e. > 4 FPKM) when co-cultured with A549 (Additional file 3: Table S1). Three of them (Afu7g04910, Afu7g06940, and Afu8g02090) were classified in at least one GO term. Additionally, 11 genes (including *cat2* and *sod3* that are related to pathogenicity) had a fold change ≥20, 54 genes (including *catA,* associated to conidial resistance to H_2_O_2_) had a fold change ≥4 and < 20, while 46 genes had a fold change ≥2 and < 4.

GO term analysis of *A. niger* showed that 127 out of 203 up-regulated genes were enriched in at least one GO term (Additional file 4: Table S2). GO terms belonging to cellular components such as cytoplasm, non-membrane-bound organelle, ribonucleoprotein complex, and ribosome were enriched in the up-regulated genes (Fig. 5). Moreover, biological process GO terms such as small molecule metabolism process, ribonucleoside biosynthesis, protein metabolism, and organic acid metabolism were enriched (Fig. 3) as well as the terms molecular function, structural molecule activity, structural constituent of ribosome, and ligase activity (Fig. 4). Twenty-one differentially expressed genes, of which 7 are classified in at least one GO term, were switched off (0 FPKM) when *A. niger* was growing in the absence of A549 but expressed (> 4 FPKM) in the presence of epithelial cells (Additional file 4: Table S2). Moreover, 37 differentially expressed genes had a fold value ≥20, 98 genes a fold change ≥4 and < 20, while 47 genes had a fold change ≥2 and < 4.

GO terms associated with cellular compartment, biological process, or molecular function were not enriched in the *A. fumigatus* and *A. niger* down-regulated genes. Of the down-regulated *A. fumigatus* genes, 47 genes encode secreted proteins, while 8 genes are part of the secondary metabolism cluster 13 (Additional file 3: Table S1). In addition, *sidD, sidJ, sidF, sidH* and *mirD* that are involved in biosynthesis, hydrolysis, and transportation of the siderophores N′,N″,N″‘-triacetylfusarinine C (TAF) and ferricrocin were down-regulated [25–29]. Three hundred two down-regulated genes were switched-off (i.e. 0 FPKM) in the presence of A549 but active (i.e. > 4 FPKM) in their absence. Moreover, 9 down-regulated genes had a fold change ≥20, including *sidD, sidJ,* and *sidF*, 48 had a fold change ≥4 and < 20, and 67 genes had a fold change ≥2 and < 4. In the case of *A. niger*, 250 genes were found to be switched-off (0 FPKM) during co-cultivation and active in the absence of A549 (> 4 FPKM) (Additional file 4: Table S2). Of the down-regulated genes, 1 had a fold change ≥20, 11 genes a fold change ≥4 and < 20, 8 genes a fold change ≥2 and < 4.

The set of differentially expressed genes of *A. fumigatus* and *A. niger* grown in the context of epithelial cells hardly shared GO terms (compare Figs. 3, 4 and 5). In fact, only 3 very broad biological processes and 1 molecular function GO terms were shared.

### Differential gene expression in A549 cells exposed to *A. fumigatus* and *A. niger*

**Fig. 6:**
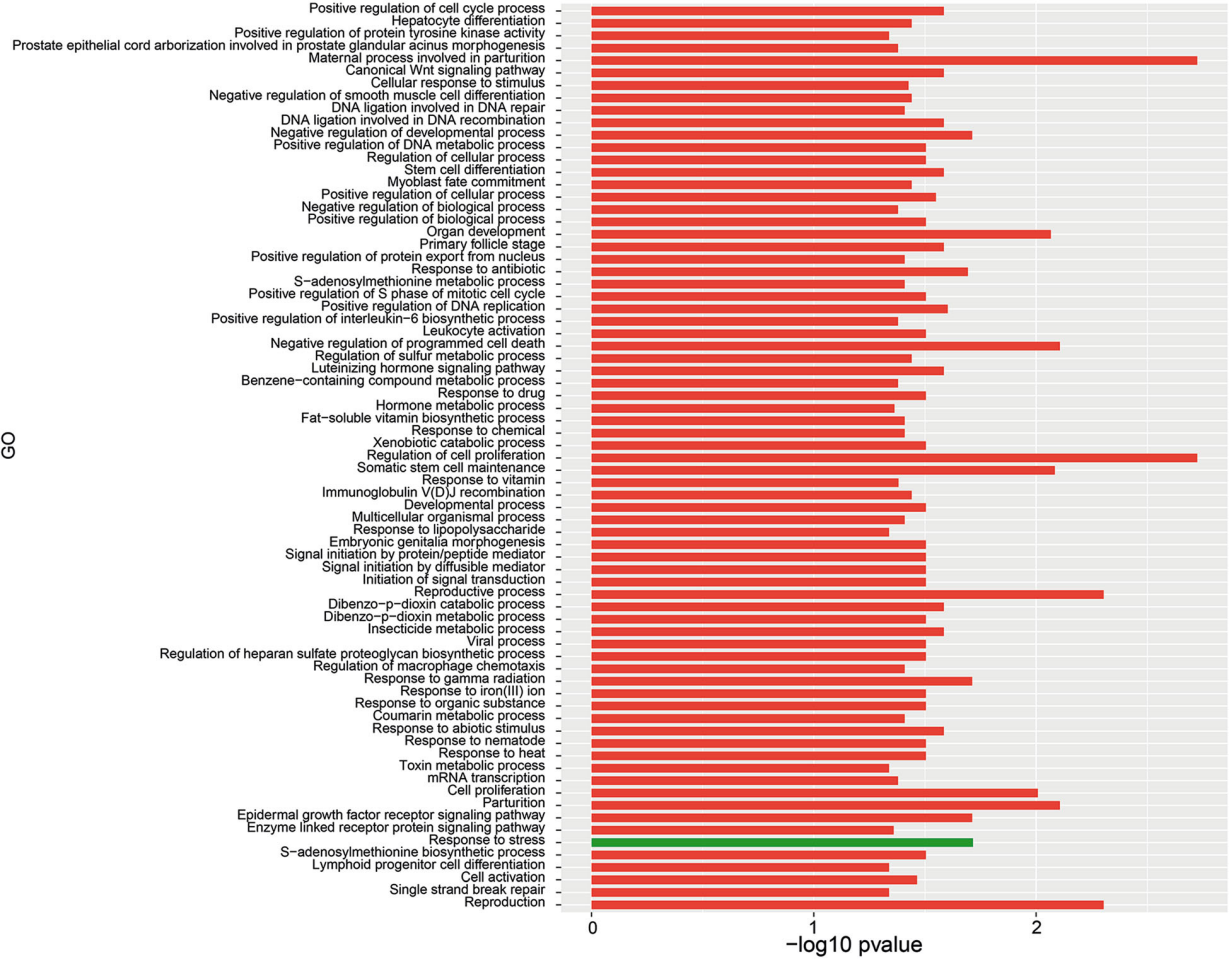
GO term enrichment analysis of biological process. A549 up-regulated (green) and down-regulated (red) genes after *A. fumigatus* co-cultivation

**Fig. 7:**
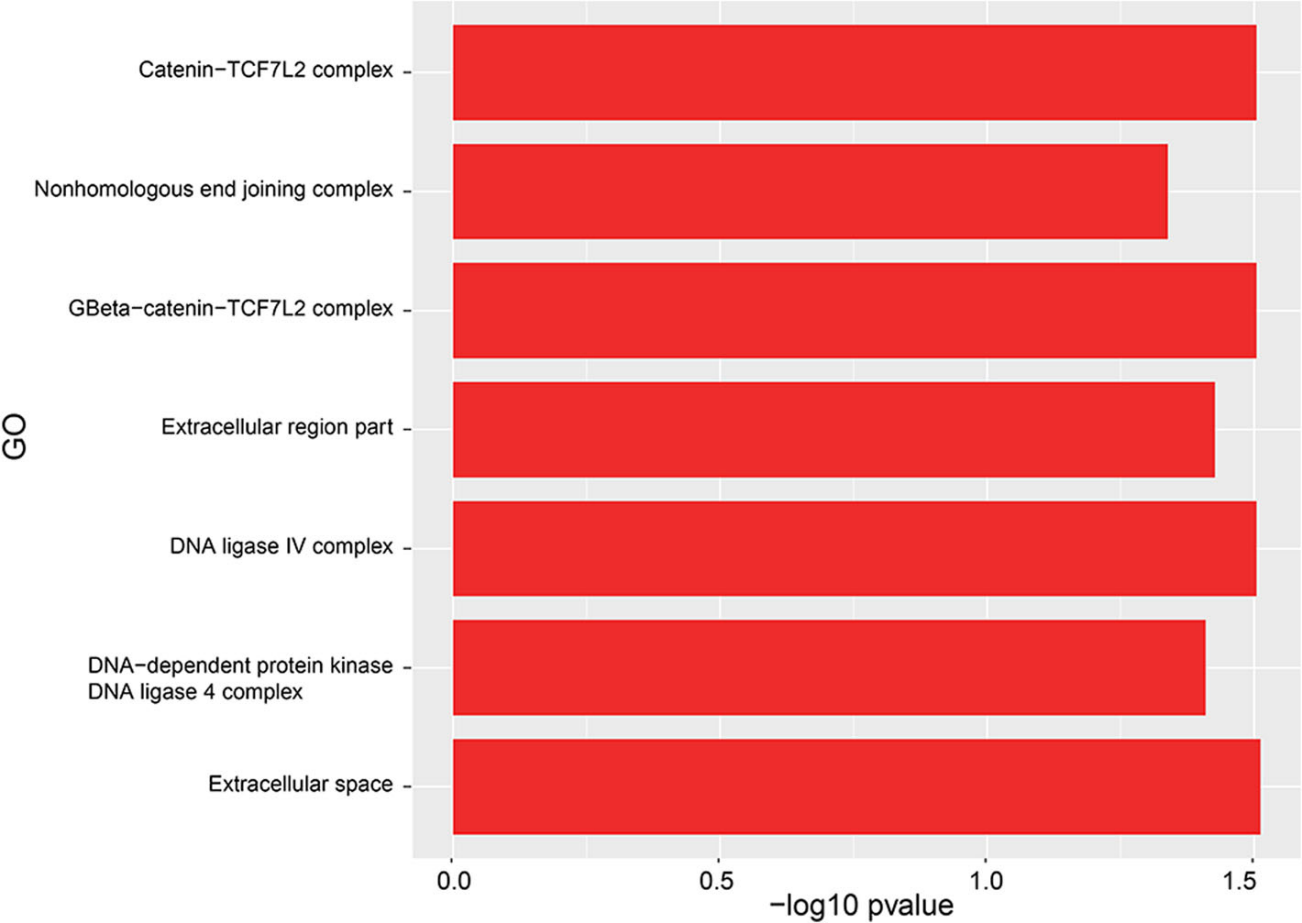
GO term enrichment analysis of cellular component. A549 down-regulated genes after *A. fumigatus* co-cultivation

**Fig. 8:**
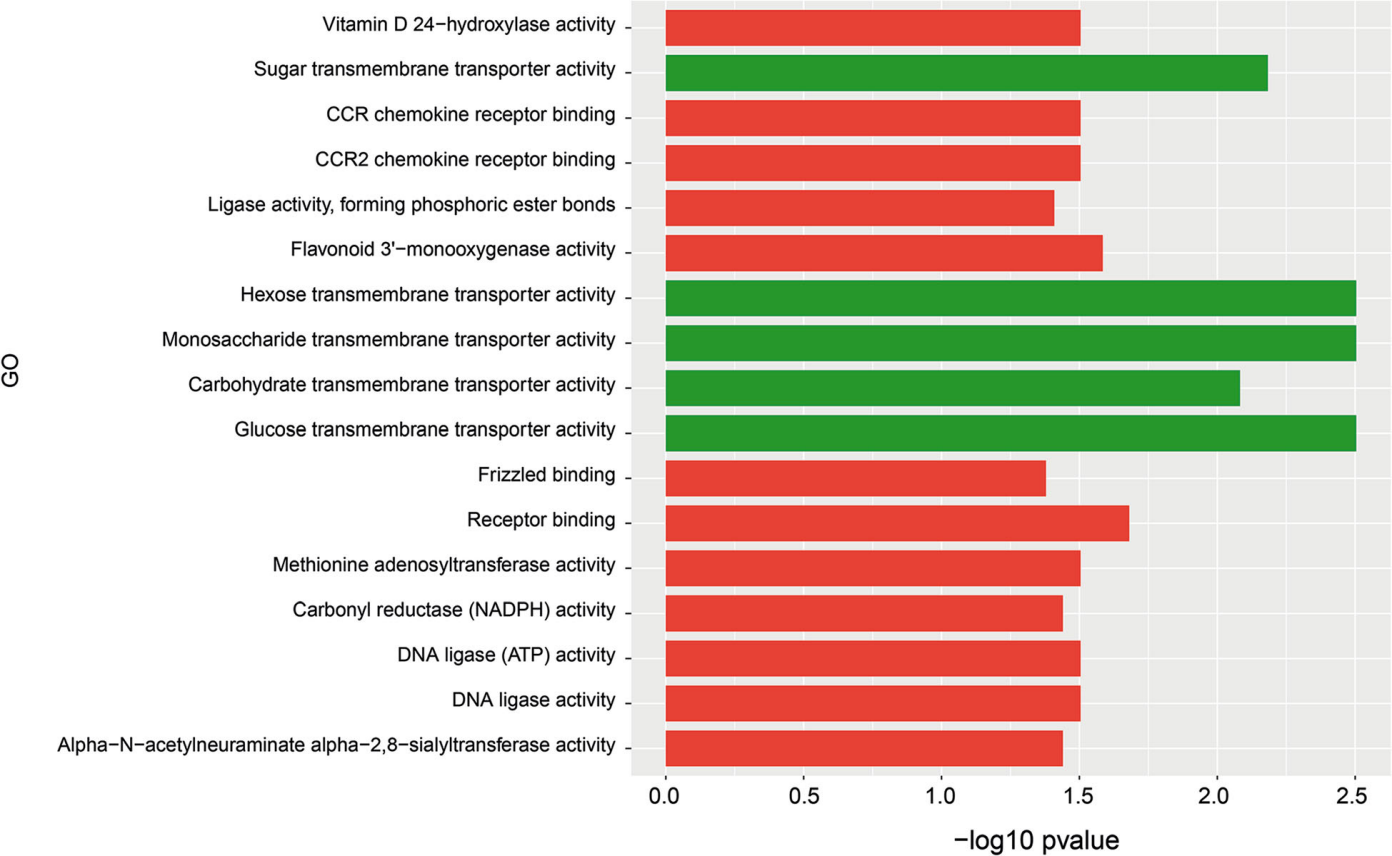
GO term enrichment analysis of molecular function A549 up-regulated (green) and down-regulated (red) genes after *A. fumigatus* co-cultivation

Co-cultivation with *A. fumigatus* resulted in only 62 and 47 up- and down-regulated A549 genes, respectively (Additional file 5: Table S3). Seventy-two biological process (Fig. 6), 7 cellular components (Fig. 7) and 12 molecular functions (Fig. 8) were enriched in the down-regulated A549 genes during *A. fumigatus* co-cultivation. Results indicate that cellular responses related to the extracellular region were down regulated after infection. Enrichment of GO terms related to CCR and CCR2 chemokine receptor binding, regulation of cell proliferation, apoptotic process, and macrophage chemotaxis, response to toxic substance, immune system development, cytokine and chemokine mediated signaling pathway in the down-regulated *A. fumigatus* genes indicate that recognition and immune response towards *A. fumigatus* were directly or indirectly repressed*.* twenty-three differentially expressed genes were switched off (0 FPKM) during co-cultivation in comparison with the control (> 0 FPKM), 3 genes had a fold change ≥20, 3 genes a fold change ≥4 < 20 and 10 genes a fold change ≥2 < 4 (Additional file 5: Table S3).

The biological process term response to stress was found up-regulated, suggesting that the infection disturbed cellular processes and cause cellular stress. The molecular terms sugar, hexose-, monosaccharide-, carbohydrate-, and glucose-transmembrane transporter activity were also up-regulated. Cellular component terms were not up-regulated. Thirty-two genes were expressed during co-cultivation (> 0 FPKM) and switched off (0 FPKM) when cells were grown in the absence of the fungus. Additionally, 4 genes showed a fold change ≥4 and 13 genes a fold change ≥2 and < 4.

Co-cultivation with *A. niger* showed 17 up- and 34 down-regulated genes (Additional file 6: Table S4). Contrary to *A. fumigatus,* GO terms were not enriched by differential expressed genes. However, 19 genes expressed in the control were found switched off after co-cultivation. From these genes, IL23A, COLEC11, PAK6, and SFTPC are known to play a role in inflammatory response, mediating endocytosis, apoptosis, and respiratory gaseous exchange, respectively [30–32]. Within the up regulated genes, tumor necrosis factor alpha induced protein 6 (TNFAIP6) was found to be active during co-cultivation while absent in the control. TNFAIP6 has been previously related to inflammatory response [33]. From the up-regulated genes, 10 genes were found switched on during co-cultivation when compared to the control, 1 gene had a fold change ≥20, 3 a fold change ≥4 and < 4 and 3 genes had a fold change ≥2 and < 4. For both *Aspergillus* species no enrichment was found using PANTHER and REACTOME.

Changes in gene expression under co-culturing conditions were validated using RT-qPCR analysis. The genes *sod3* and *dprB* of *A. fumigatus*, An903 and An693 of *A. niger* were confirmed to be upregulated in these transcriptomic analysis (Table 4).

**Table 4:** Validation RNA sequencing result by RT-qPCR in fungi

	Gene	Expression	*P* value		Log fold change RNA-seq
*A. fumigatus*	sod3	6.207	0.034	UP	52.4
	dprB	15.455	0.000	UP	33
*A. niger*	An903	27.901	0.000	UP	49.8
	An693	8.927	0.000	UP	87.1

### Effects of tween and MOI on IL-8 regulation

In this study we did not observe an upregulation of immune response genes like IL-8 in contrast to previous reports [17, 21]. We noticed that in contrast to these reports we did not use Tween to harvest conidia. Tween was previously suggested to induce immune response [34] and we therefore studied whether a 12 h exposure of A549 cells to different concentrations of Tween 20 or Tween 80 resulted in upregulation of IL-8 using RT-qPCR (Additional file 7: Table S6A). Indeed, significant upregulation of IL-8 was observed when concentrations of Tween 20 or Tween 80 were 0.05% but not at lower concentrations indicating that this cannot explain the differences between our results and those reported previously [17, 21]. Another difference with previous studies was the high multiplicity of infection (MOI) and the absence of a wash step to remove the unassociated conidia during co-cultivation with epithelial cells. We therefore repeated our co-culturing experiments at a much higher MOI (approximately 1) resulting on average in 1 conidia/A549 cell and, in addition, now used also *A. fumigatus* and *A. niger* conidia isolated with saline with or without 0.1% Tween 20. Conidia harvested with Tween were washed with saline before use. Subsequent co-incubation was performed for a total 8 h (without wash step) with A549 cells but no upregulation of IL-8 was observed at this time point as determined by RT-qPCR with conidia isolated with or without Tween-20 (Additional file 7: Table S6B). Interestingly, similar co-incubations of conidia (harvested either with or without Tween 20) with epithelial cells for 12 h at a high MOI (approximately 1 conidia/A549 cell), but with a wash step to remove non-associated conidia, did result in upregulation of IL-8 for both fungal species (Additional file 7: Table S6C). The expression of genes EGR1, TNFAIP6 and FSIP1 were validated by RT-qPCR. These genes were found upregulated in A549 cells as observed in our transcriptomic data (12 h time point). We already detected upregulation of these genes after 8 h although to a lesser extend as compared to 12 h (Additional file 7: Table S6B and C).

## Discussion

*A. fumigatus* is more efficiently internalized by A549 and shows delayed germination when compared to *A. niger.* Moreover, *A. fumigatus* grows parallel to the epithelium, whereas *A. niger* grows perpendicular to this cell layer [18]*.* Here, RNAseq was performed to study the response of *A. fumigatus* and *A. niger* during late stages of infection initiation upon exposure at an MOI of approximately 0.1 to A549 cells. At this stage part of the conidia still have not germinated, while others have started to form hyphal structures that grow inside this type II epithelial lung cells or even might have escaped the cells. Expression profiles were indeed indicative for the presence of different fungal morphotypes. Genes, *sod3*, *trr1, cycA* and *gel4* (Afu2g05340) are known to be up-regulated during germination [35–38], *pst2, cat2,* Afu4g08240, Afu4g12590, Afu6g13330, and Afu7g06770 encode conidia-enriched proteins [39–41], while transcripts of *scf1, bipA,* and *dprA* are exclusively present in dormant conidia [42–44]. A comparison of our RNA-seq results with the ones of Hagiwara et al. 2016 showed that *A. fumigatus* and *A. niger* have different distributions of conidia associated genes (CAGs) and germination associated genes (GeAGs) [45]. A total of 74 out of 687 CAGs were differentially expressed in *A. fumigatus* while this was true for 46 out of 694 CAGs of *A. niger*. In contrast, 47 out of 687 and 85 out of 1241 GeAGs were differentially expressed in *A. fumigatus* and *A. niger* respectively (Table 5). Genes that might have a role in pathogenesis were found in the CAGs category for *A. fumigatus* but not in *A. niger*; for example *catA* (Afu6g03890) was found to be upregulated duing infection. This gene is dependent on the expression of AtfA (Additional file 8: Table S5), a transcripton factor involved in regulation of dormancy (Hagiwara et al. [45]). Our results indicate that a larger proportion of *A. fumigatus* conidia remained in a dormant stage in the presence of A549 cells as compared to *A. niger*, and together with an intracellular localization is favoring immune evasion.

**Table 5:** Number of conidia associated genes (CAGs) and germination associated genes (GeAGs) (according to Hagiwara et al [45]) in *A. fumigatus* and *A. niger* in the presence of A549 epithelial cells

	CAGs	GeAGs
*A. fumigatus*	74	47
*A. niger*	46	85

Differentially expressed genes of both species only share 4 GO terms that are enriched in the up- and down-regulated genes and only show an overlap of 53 genes. A549 cells also respond differently to both fungi. For instance, down-regulated genes of A549 cells are enriched in GO-terms related to the immune response, while this was not observed in the case of *A. niger.*

Genes described to benefit fungal survival were found up-regulated in *A. fumigatus* when exposed to A549 cells*.* For instance, a large number of genes induced under hypoxia conditions (*cox5b*, *cycA, rip1*, *gel4,* Afu3g06190, and Afu1g10780) and stress response (*scf1, dprA,* and *dprB*) were found upregulated. Apparently, *A. fumigatus* senses and responds to stress conditions and low levels of oxygen. In the case of *A. niger,* only the orthologues of *A. fumigatus rip1* and Afu1g10780 were found up-regulated. Genes *sod3, cat2,* and *catA* that encode a dismutase and catalases, respectively, were also up-regulated in *A. fumigatus*. These proteins protect the fungus against oxidative damage [35, 46, 47]. In contrast, homologous of these genes were not found differentially expressed after *A.niger* infection. GO terms involved in iron metabolism and binding were also enriched in the differentially expressed up-regulated genes of *A. fumigatus*. Iron acquisition is essential for *A. fumigatus* growth in human cells [48]. We found up-regulation of *sdh2* (Afu5g10370), involved in iron homeostasis and previously described to be expressed after neutrophils exposure [41]. However, genes of the siderophore biosynthesis pathway (*sidA*, *sidC*, *sidF*, *sidD*, and *mirD*) were found down-regulated after co-cultivation. This suggests that alternatives pathways are being used for iron acquisition. Alternatively, iron requirements are less strong at the late stage of infection initiation. Neither *sdh2* nor the orthologues siderophore pathway genes *sidA, sidC, sidF,* and *sidD* were differentially expressed in *A. niger.* However, 5 genes involved in iron binding, transportation, and homeostasis were found down-regulated indicating that these two fungal species adapt differently to iron limitation in these conditions. Only secretome and secondary metabolites annotated terms were enriched in the down-regulated *A. fumigatus* genes. Notably, some genes related to pathogenesis were found down-regulated or even switched off. For example, *sidA* (see above) and *gpgA* that encodes a G protein-coupled receptor required for gliotoxin production [49, 50] were down-regulated. This supports our previous findings that *A. fumigatus* infection occurs in a quiet and silent way, possibly avoiding immune response [18]. The response of *A. niger* upon interaction with A549 is more difficult to interpret. Orthologues of the differentially expressed *A. fumigatus* pathogenicity genes were not found in *A. niger*. However, the fact that GO terms and genes associated with hydrolase activity, proteolysis, and iron binding were found up-regulated shows that *A. niger* does adapt and respond to its host [51, 52].

Gene expression analysis of A549 cells revealed different transcriptomic profiles after co-cultivation with *A. fumigatus* and *A. niger.* GO terms related with immune response were down-regulated upon exposure to *A. fumigatus* but not in the case of *A. niger.* This indicates that the former fungus reprograms A549 not to be fully immunologically alert. For example, down regulation of cytokine and chemokine release by epithelial cells would repress recruitment of phagocytes [53]. Strikingly, co-cultivation resulted in down regulation of beta-catenin TCF7L2 complex, a protein complex that contains beta-catenin and the transcription factor TCF7L2 that is also known as TCF4. The multifunctional beta-catenin protein functions in cell-cell adhesion by binding cadherins. It also binds to the Wnt signal transduction pathway and a link with regulation of innate immunity in lung cells was recently reported [54, 55]. This suggests that homeostasis of A549 cells as well as other processes regulated by the Wnt/beta-catenin pathway such anti- or proinflamatory cytokines release, might been affected after *A. fumigatus* infection.

Co-cultivation of epithelial cells with *A. niger* resulted in upregulation of the gene encoding TNFAIP6 or TNF-α-induced protein 6 (TSG-6). TSG-6 is a 35 kDa secreted protein and is a potent anti-inflammatory factor. Upregulation of this gene was previously reported to be involved in attenuating lung cell injury by lipopolysaccharide-induced acute lung injury [33]. This suggests that upregulation of TSG-6 is a result of epithelial lung cell damage induced by *A. niger*, which is not observed with *A. fumigatus*. Together, these results show a marked difference in life style between *A. fumigatus* and *A. niger* when grown in the presence of type II epithelial cells.

Other studies have described gene expression profiles from airway epithelial cells and *A. fumigatus* strain ATCC 13703 but in these cases expression was studied 6 h after co-cultivation [16, 17]. One study revealed that 889 genes of immortal bronchial epithelial 16HBE14o-cells were differentially expressed when exposed to *A. fumigatus* conidia for 6 h. Genes associated with repair and inflammatory processes such as matrix metalloproteinases, chemokines, and glutathione S-transferase where found up-regulated [16]. In another study 255 genes of the cell line were differentially expressed. Among the most highly enriched up-regulated functional gene classes were groups involved in innate immune response, defense response, and the inflammatory response. None of these GO terms where found up-regulated in A549 cells after co-cultivation *A. fumigatus* and *A. niger* in our study. In contrast, GO terms related with response to external stimuli such as temperature, toxins, and abiotic factors were down-regulated after co-cultivation with *A. fumigatus.* On the other hand, 150 and 33 *Aspergillus* genes were found up- and down regulated respectively, after 6 h of co-cultivation with 16HBE14o- cells [17]. Different to our study, genes involved in vacuolar acidification and metallopeptidase activity but also upregulation of iron siderophore biosynthesis was observed. The differences between these studies and ours are not easily explainable but could be related to differences in the cell lines and the *A. fumigatus* strains used. Oosthuizen et al., reported differences when comparing gene expression with RT- qPCR between two different cell types [17]. In addition, the magnitude of the fold change is modest as compared to our results where fold changes are much larger. Furthermore, Chen et al., reported a transcriptome profile of human lung epithelial cells A549 interacting with *A. fumigatus* strain B5233 and observe IL-8 induction after 8 h of co-incubation with A549 cells*.* There are various differences between these experiments as compared to ours, one relates to the use of Tween during harvesting of the fungal conidia used for the exposure studies and the other to the MOI and the absence of a wash step to remove the unassociated conidia during co-culturing of cells with fungi [17, 21]. In our initial experiments we used a relative low MOI of approximately 0.1 resulting in 0.06 conidia/A549 cell. The amount of conidia used during co-cultivation and a wash step after 2 h might be crucial since conidia germinating extracellularly might trigger the immune response. In contrast to our study, Oosthuizen et al., and Chen et al., used 0.1% Tween 20 to isolate the fungal conidia and which were washed before use in order to remove the detergent [17, 21]*.* Tween 20 shares some structural similarity to Lipid A of Gram-negative bacteria, especially the short saturated fatty acids and has been implicated to affect the immune response and *reactive oxygen species* (ROS) production [34]. Tween 20 contains lauric acid that might be released and affect Tol-like receptors and induce NF-κB activation [56, 57]. We demonstrate here that Tween does not play a role in the induction of IL-8 expression but that this is related to the MOI. In addition to differences in experimental set up, we cannot rule out that differences in *A. fumigatus* strains used can affect experimental outcomes. Indeed, Watkins et al. showed that a comparative transcriptomic analysis between the *A. fumigatus* strains Af293 and CEA10 during a 6 or 16 h infection of A549 cells did not result in a conserved transcriptional response of *A. fumigatus* [58]. It should be noted however that in this latter study no wash step was introduced to remove non-associated conidia which resulted by 16 h in a A549-cell monolayer covered with a hyphal mat. Our results obtained with a high MOI (of 1) indicate that induction of the immune response (as monitored by IL-8 expression) can be the result of increased contact between germinated hyphae and/or products produced by these hyphae with the epithelial cells being reminiscent to recent studies with *Candida albicans* [59]. In contrast to other studies we included a wash step after 2 h to remove excess of non-associated conidia. We consider this step very crucial since it allows a detailed transcriptomic analysis of the associated conidia in the context of the epithelial cells specifically. The transcriptomic analysis at low MOI shows clear differences in life style between the studied fungi as well as the response of the epithelial cells, amongst others down regulation of cytokine- and chemokine signaling events due to *A. fumigatus*.

## Conclusions

Our study revealed that expression profiles of *A. fumigatus* and *A. niger* differed drastically after 12 h upon contact with A549 cell. Repression (or delay) of germination and a predominant conidial stage appears to be crucial for *A. fumigatus* to limit immune response and prolong its survival. It is estimated that humans inhale around 200 to 300 conidia per day, most of these are cleared by pulmonary defense mechanisms. This implies that in nature, *Aspergillus* infections develop from a very limited number of conidia per surface area of lung tissue, rather than from a large amounts of conidia per lung cell. Our results showed that the activation of immune response by lung epithelial cells is also dependent on the amount of conidia per lung cell. Upregulation of pro-inflammatory cytokine IL-8 was observed at high MOI (approximately 1) for both fungal species, but not at low MOI (approximately 0.1). Under these latter condition, genes associated with immune response including genes involved in cytokine and chemokine release were especially down regulated by *A. fumigatus*. Furthermore, under low MOI conditions, genes previously associated with pathogenicity involved in stress response and oxidative damage were found upregulated in *A. fumigatus* but not in *A. niger.* Together, our work highlights main differences in gene regulation between the host and two different *Aspergillus* strains (non-pathogenic *A.niger* and the pathogenic *A. fumigatus*), which is affected in part by the amount of conidia per lung cell.

## Additional files

Additional file 1: **Table S7.** Overview of RNA-seq mapping of reads per sample. Electronic format. (XLSX 8 kb)

Additional file 2: **Figure S1.** Overview of the output metrics from CuffLinks Pipeline. Electronic format: A. *Aspergillus fumigatus*; A1: Dispersion graph; A2: FPKM Density; A3: PCA per sample; A4: PCA; B. *Aspergillus niger*; B1: Dispersion graph; B2: FPKM Density; B3: PCA per sample; B4: PCA; C: A549 cells co-cultivated with *A. fumigatus*; C1: Dispersion graph; C2: FPKM Density; C3: PCA per sample; C4: PCA; D: A549 cells co-cultivated with *A. niger*; D1: Dispersion graph; D2: FPKM Density; D3: PCA per sample; D4: PCA. (PDF 69372 kb)

Additional file 3: **Table S1.** Differentially gene expression and enriched GO terms of *A. fumigatus* after 12 h of co-cultivation with A549 cells. Electronic format. (XLSX 63 kb)

Additional file 4: **Table S2.** Differentially gene expression and enriched GO terms of *A. niger* after 12 h of co-cultivation with A549 cells. Electronic format. (XLSX 38 kb)

Additional file 5: **Table S3.** Differentially gene expression and enriched GO terms of A549 cells after 12 h of co-cultivation with *A. fumigatus.* Electronic format. (XLSX 29 kb)

Additional file 6: **Table S4.** Differentially gene expression and enriched GO terms of A549 cells after 12 h of co-cultivation with *A. niger.* Electronic format. (XLSX 17 kb)

Additional file 7: **Table S6.** Gene expression analysis using RT-qPCR. Electronic format. (DOCX 15 kb)

Additional file 8: **Table S5.** Conidia associated genes (CAGs) and germination associated genes (GeAGs) found in *A. fumigatus* and *A. niger* after 12 h of co-cultivation with A549 cells. Electronic format. (XLS 83 kb)

## Abbreviations

CAGs: conidia associated genes
DMEM: Dulbecco’s Modified Eagle’s Medium
GeAGs: germination associated genes
GO: Gene Ontology
MDB: membrane desalting buffer
MFS: major facilitator superfamily
MOI: multiplicity of infection
PDA: potato dextrose agar
ROS: *reactive oxygen species*
SRA: sequence read archive
TAF: N′,N″,N″‘-triacetylfusarinine C
TNFAIP6: tumor necrosis factor alpha induced protein 6
TSG-6: TNF-α-induced protein 6
