# Supplementary material for: Expression profile analysis reveals that Aspergillus fumigatus but not Aspergillus niger makes type II epithelial lung cells less immunological alert
Source: BMC Genomics. 2018 Jul 13;19:534. doi: 10.1186/s12864-018-4895-3 (PMC6044037; doi:10.1186/s12864-018-4895-3)
Supplement: Supplementary file 2 — Figure S1. Overview of the output metrics from CuffLinks Pipeline. Electronic format: A. Aspergillus fumigatus; A1: Dispersion graph; A2: FPKM Density; A3: PCA per sample; A4: PCA; B. Aspergillus niger; B1: Dispersion graph; B2: FPKM Density; B3: PCA per sample; B4: PCA; C: A549 cells co-cultivated with A. fumigatus; C1: Dispersion graph; C2: FPKM Density; C3: PCA per sample; C4: PCA; D: A549 cells co-cultivated with A. niger; D1: Dispersion graph; D2: FPKM Density; D3: PCA per sample; D4: PCA. (PDF 69372 kb) [file 12864_2018_4895_MOESM2_ESM.pdf]

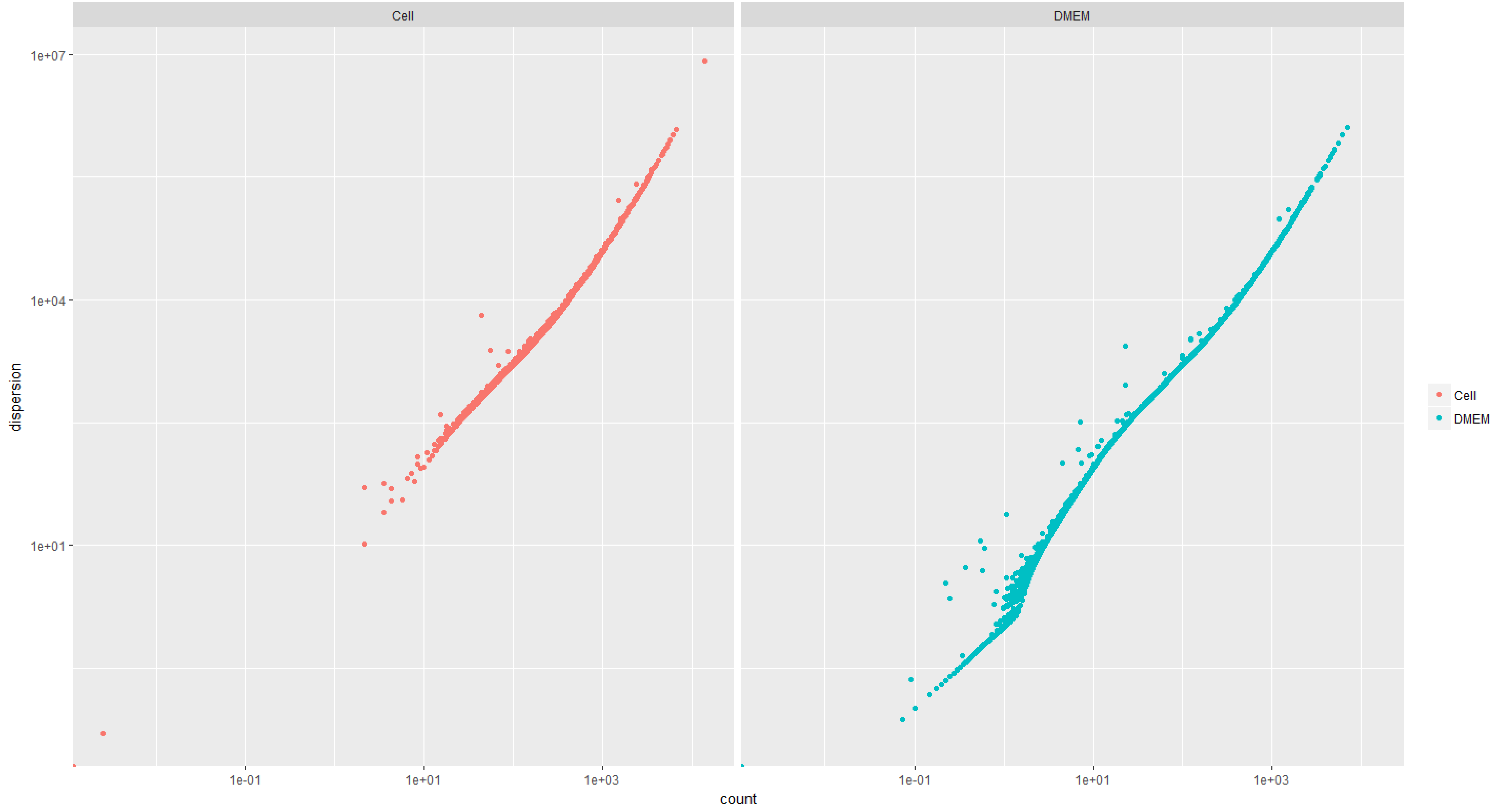

A1: Dispersion graph from *A. fumigatus* in co-culture with A549 cell (Cell) and DMEM

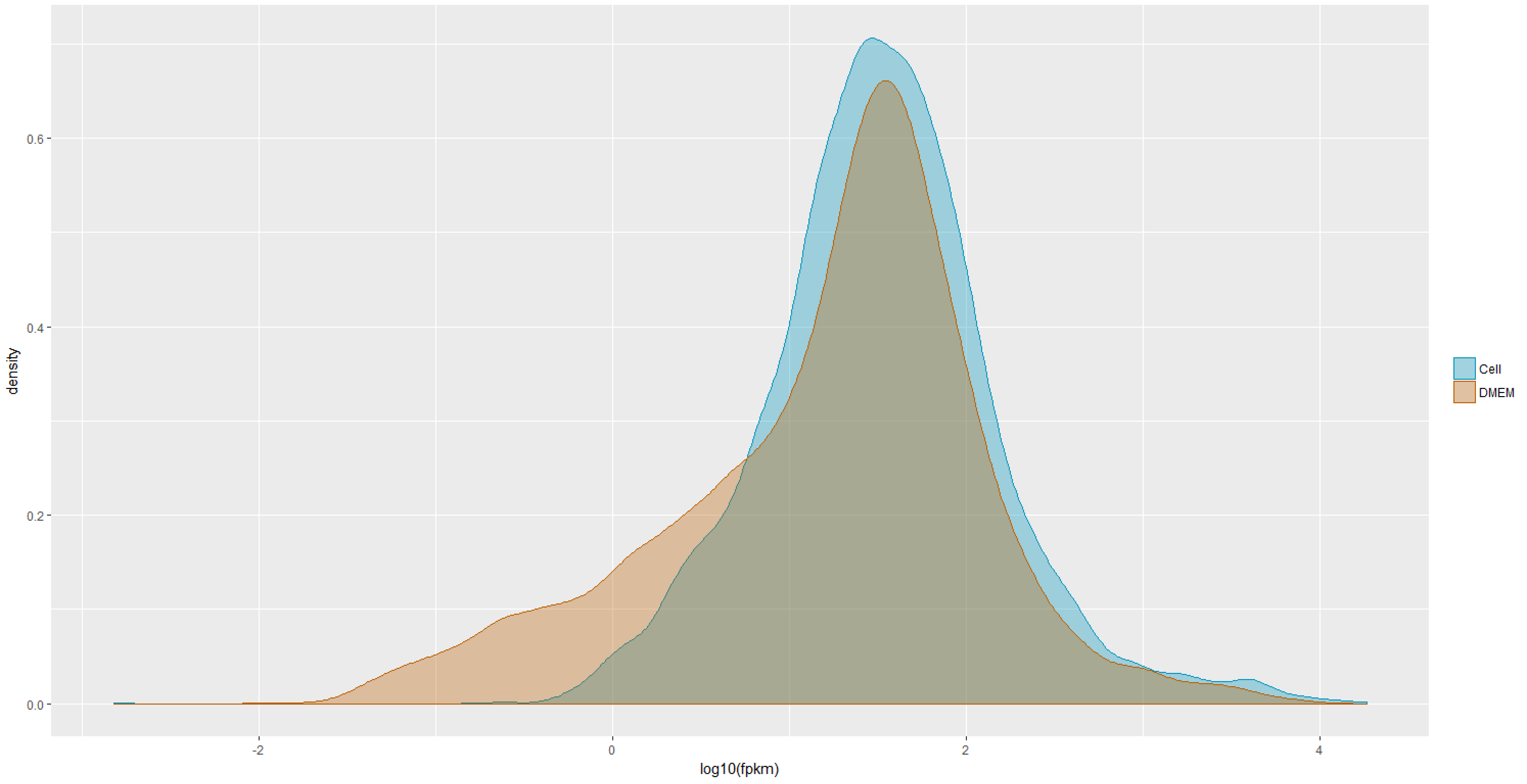

A2: Expression level distribution of mapped genes in *A. fumigatus* in co-culture with A549 cell (Cell) and DMEM. FPKM= fragments per kilobase of transcript per million fragments mapped.

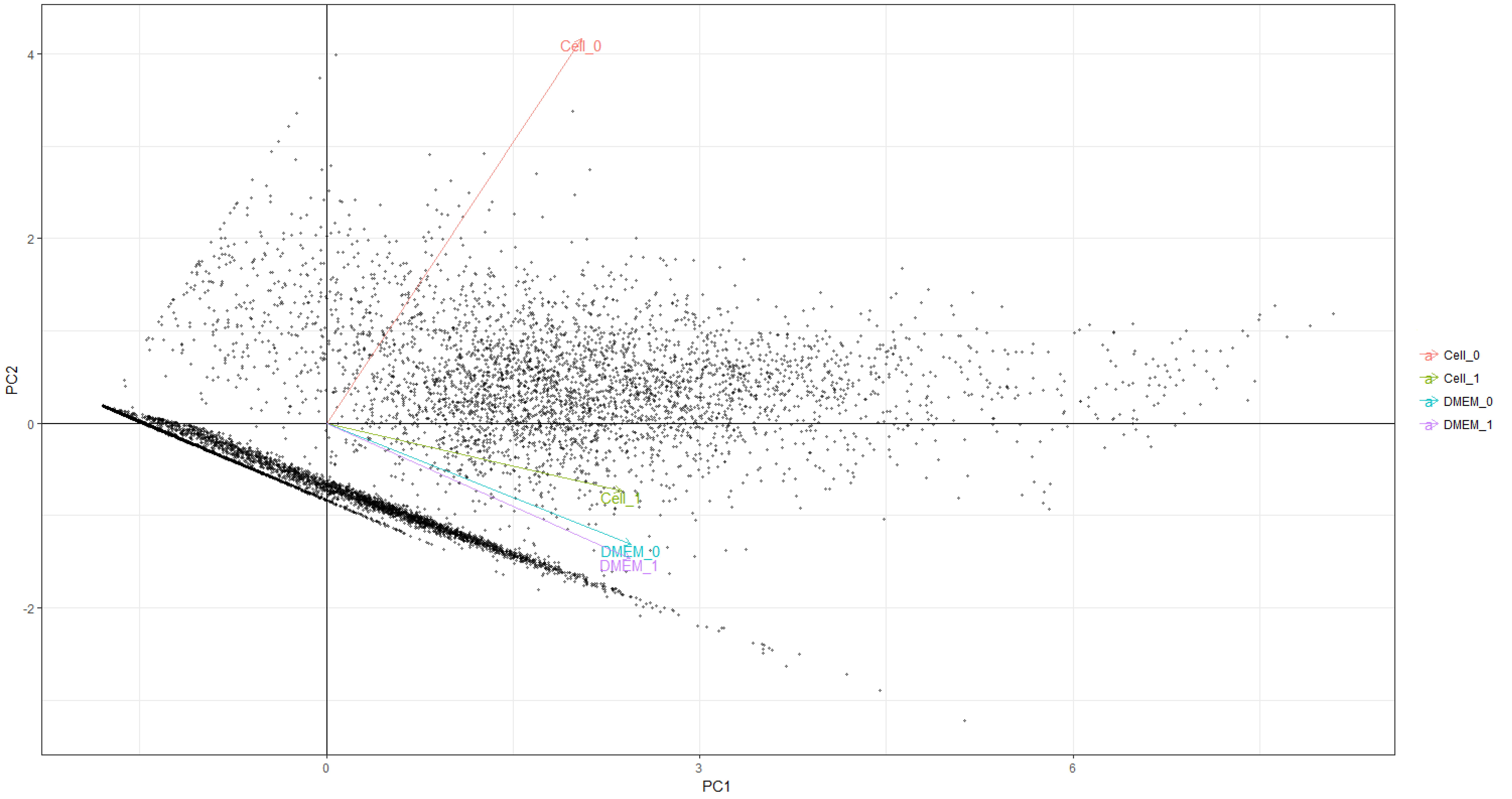

A3: Principal component analysis per sample of RNA-seq data from *A. fumigatus* in co-culture with A549 cell (Cell) and DMEM.

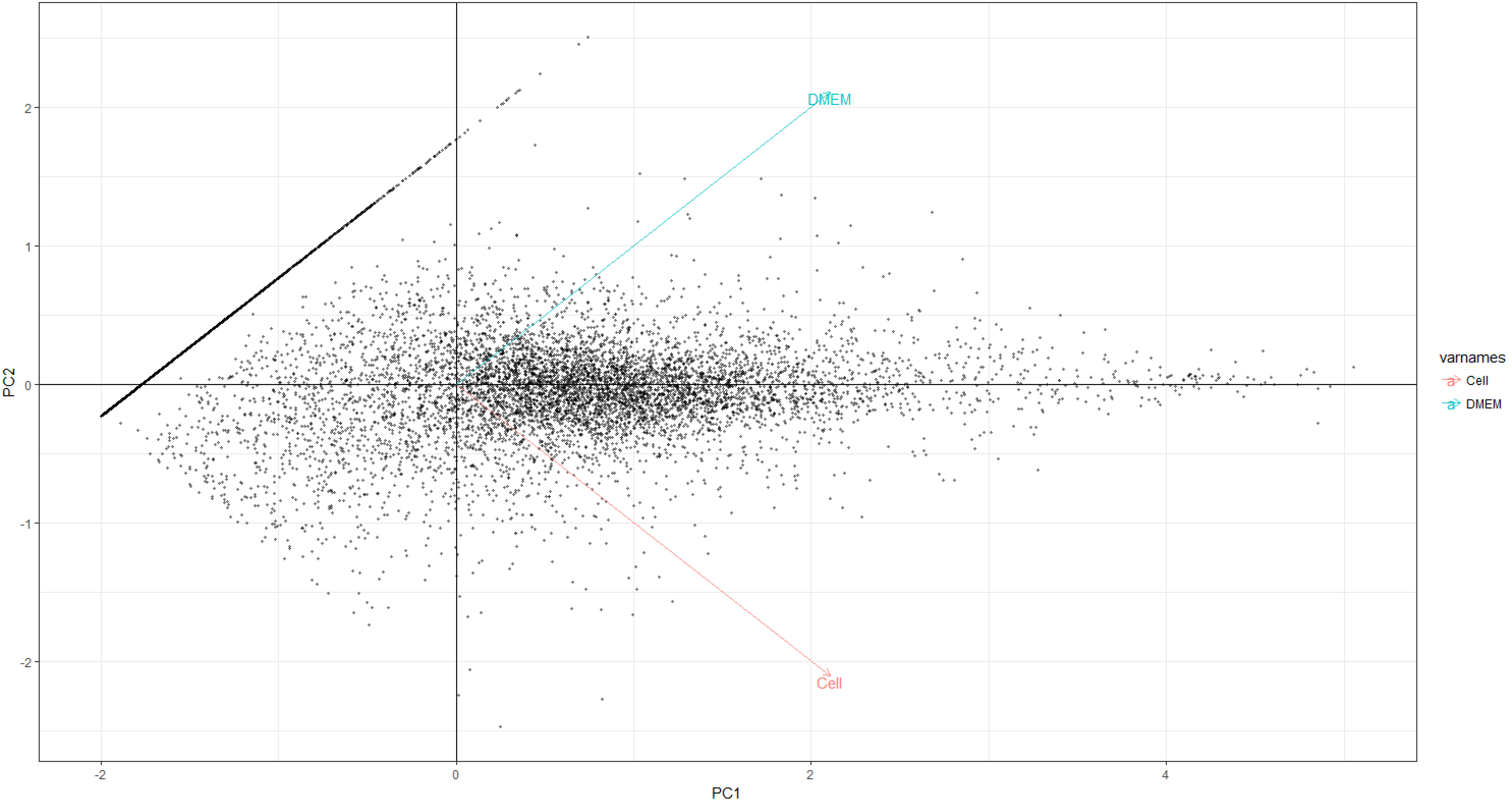

A4: Principal component analysis of RNA-seq data from *A. fumigatus* in co-culture with A549 cell (Cell) and DMEM

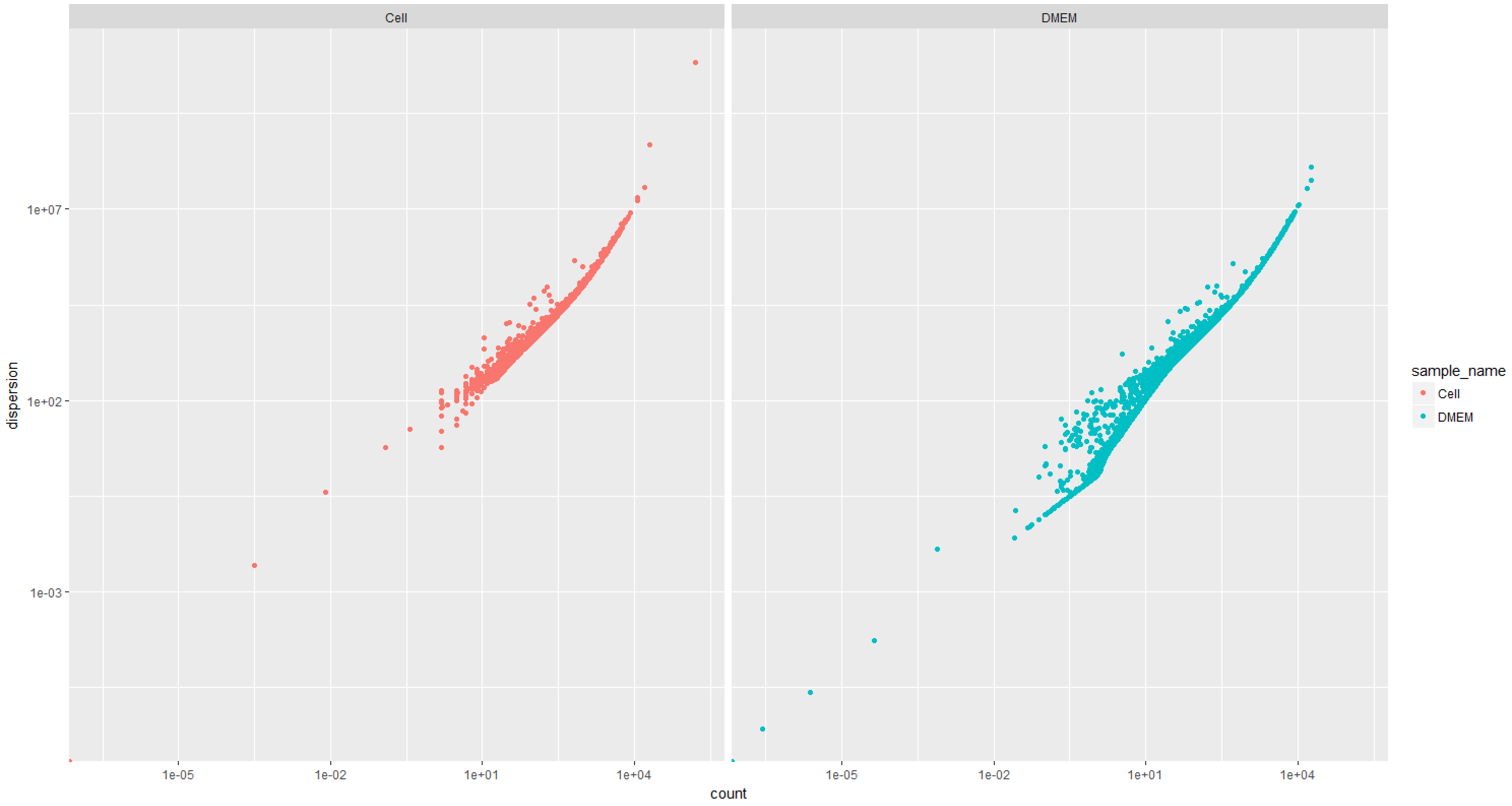

B1: Dispersion graph from *A. niger* in co-culture with A549 cell (Cell) and DMEM.

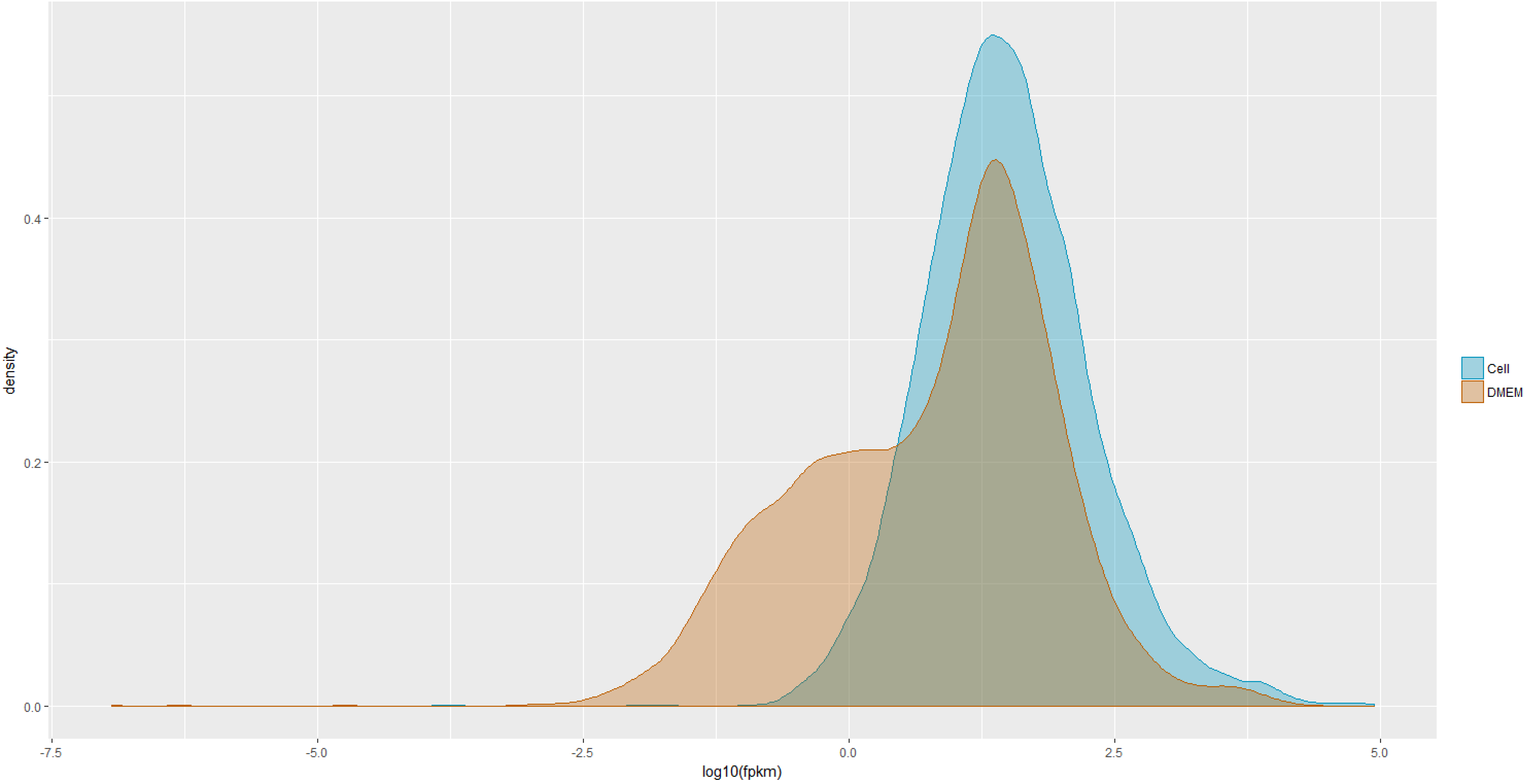

B2: Expression level distribution of mapped genes in *A. niger* in co-culture with A549 cell (Cell) and DMEM. FPKM= fragments per kilobase of transcript per million fragments mapped.

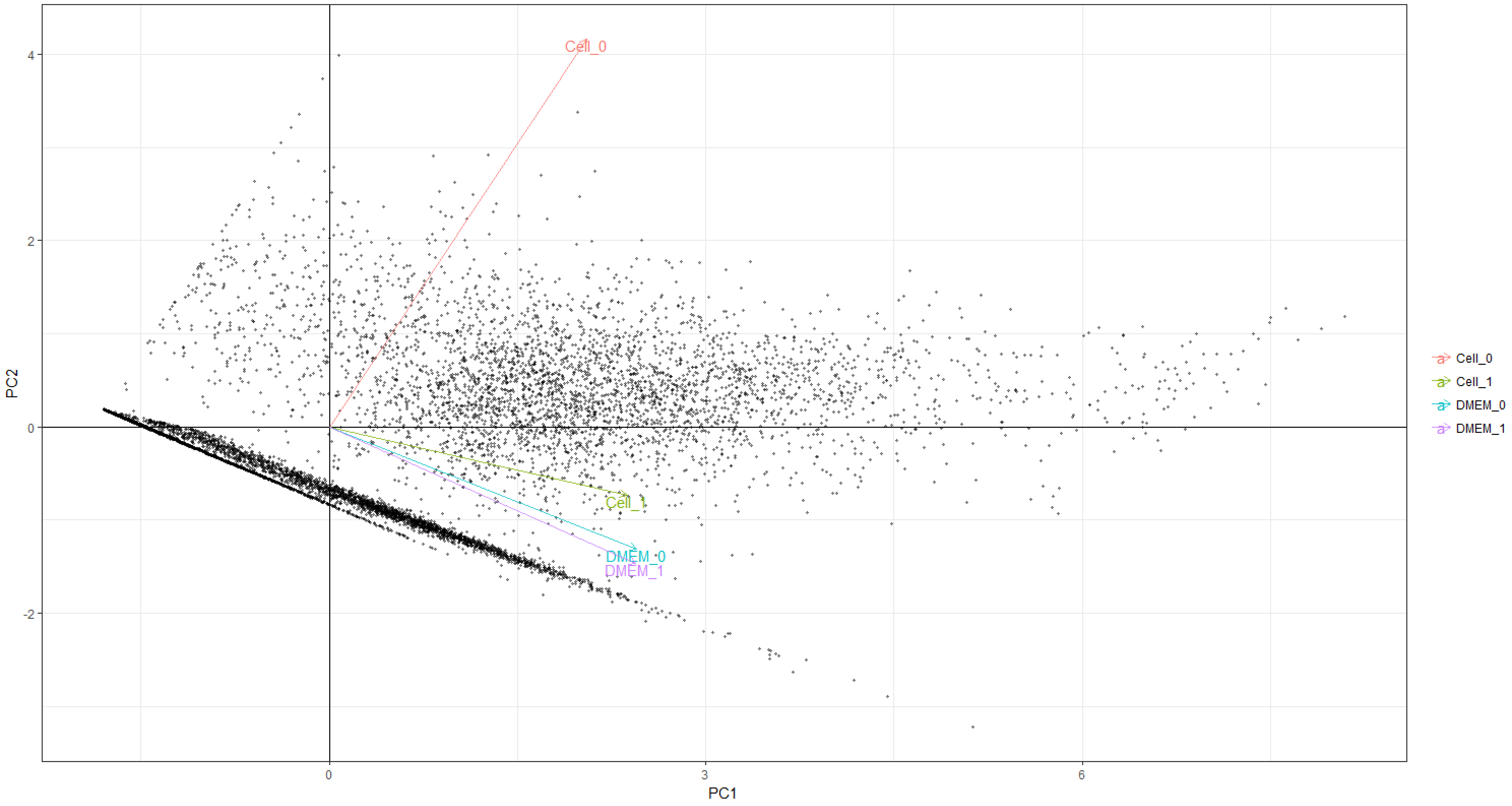

B3: Principal component analysis per sample of RNA-seq data from *A. niger* in co-culture with A549 cell (Cell) and DMEM.

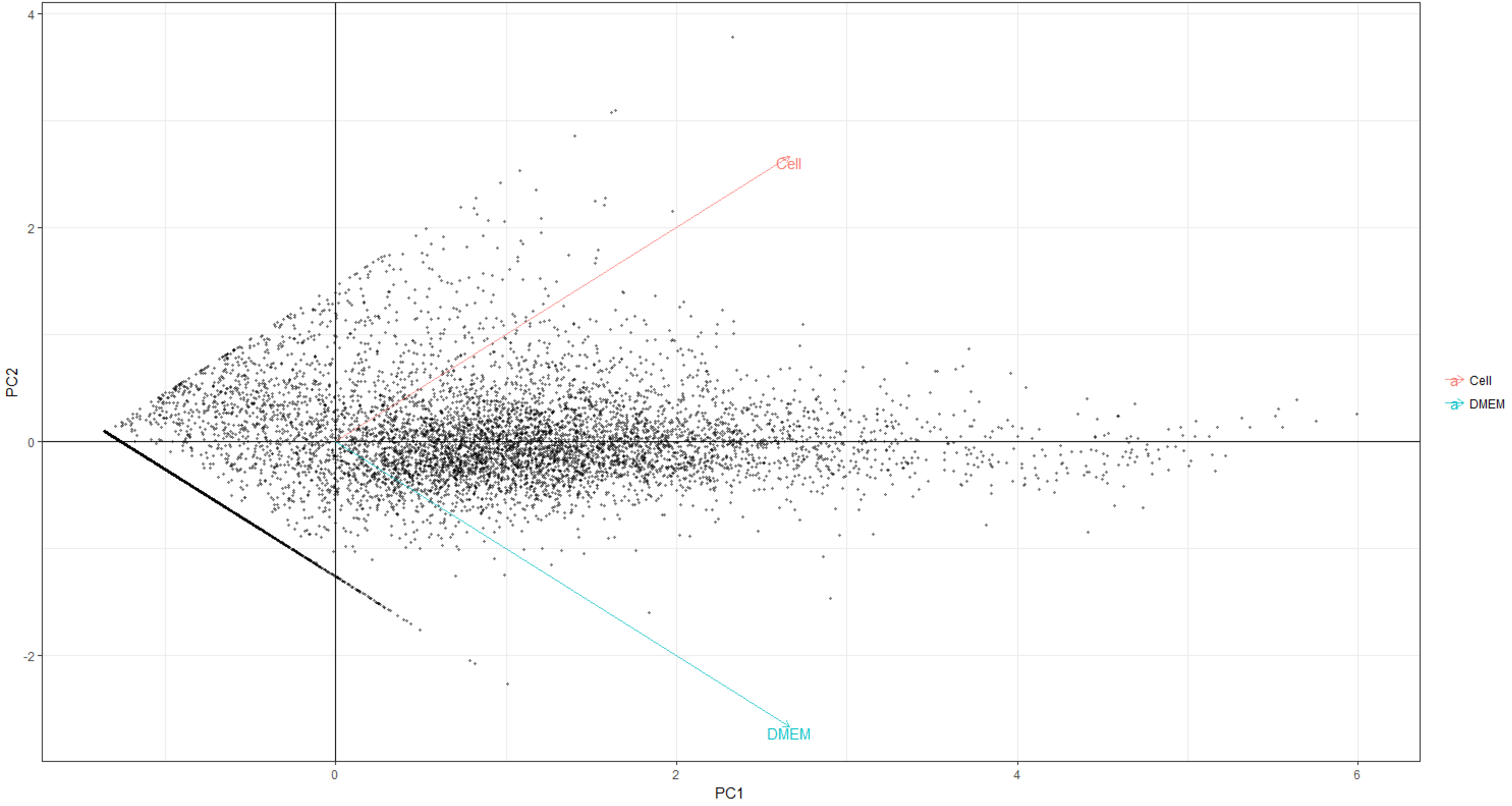

B4: Principal component analysis of RNA-seq data from *A. niger* in co-culture with A549 cell (Cell) and DMEM.

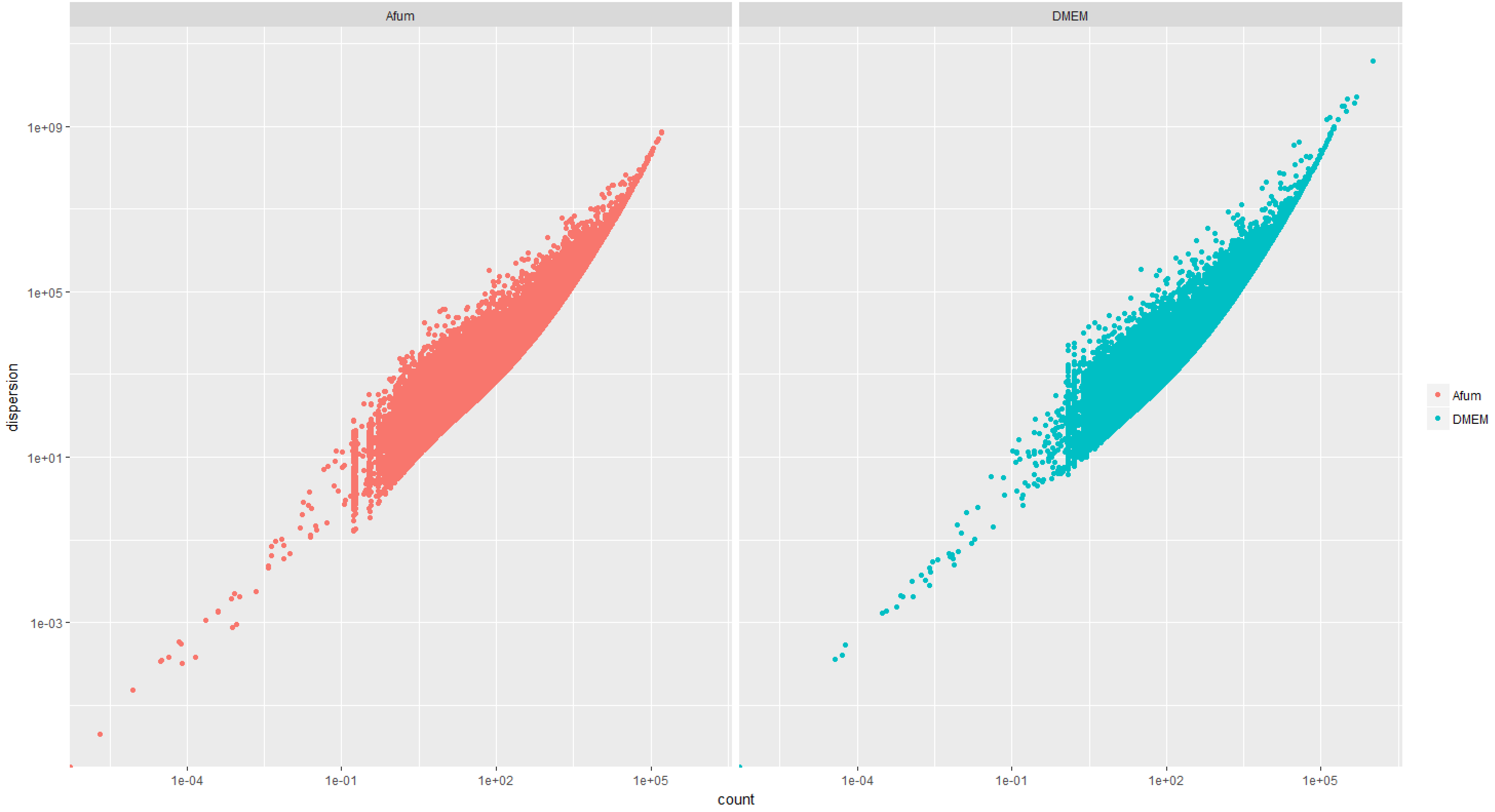

C1: Dispersion graph from A549 cells in co-culture with *A. fumigatus* (Afum) and DMEM.

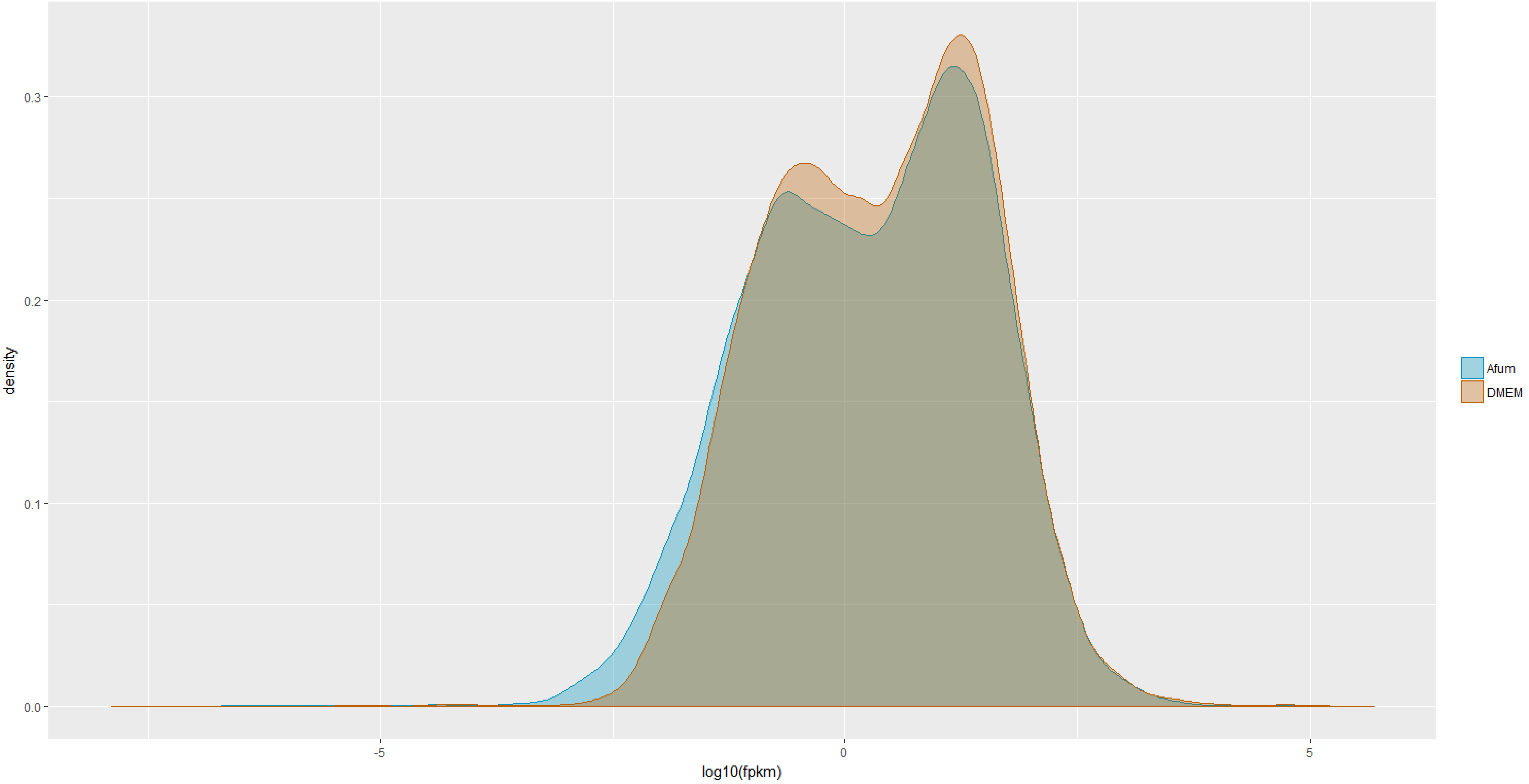

C2: Expression level distribution of mapped genes in A549 cells in co-culture with *A. fumigatus* (Afum) and DMEM. FPKM= fragments per kilobase of transcript per million fragments mapped.

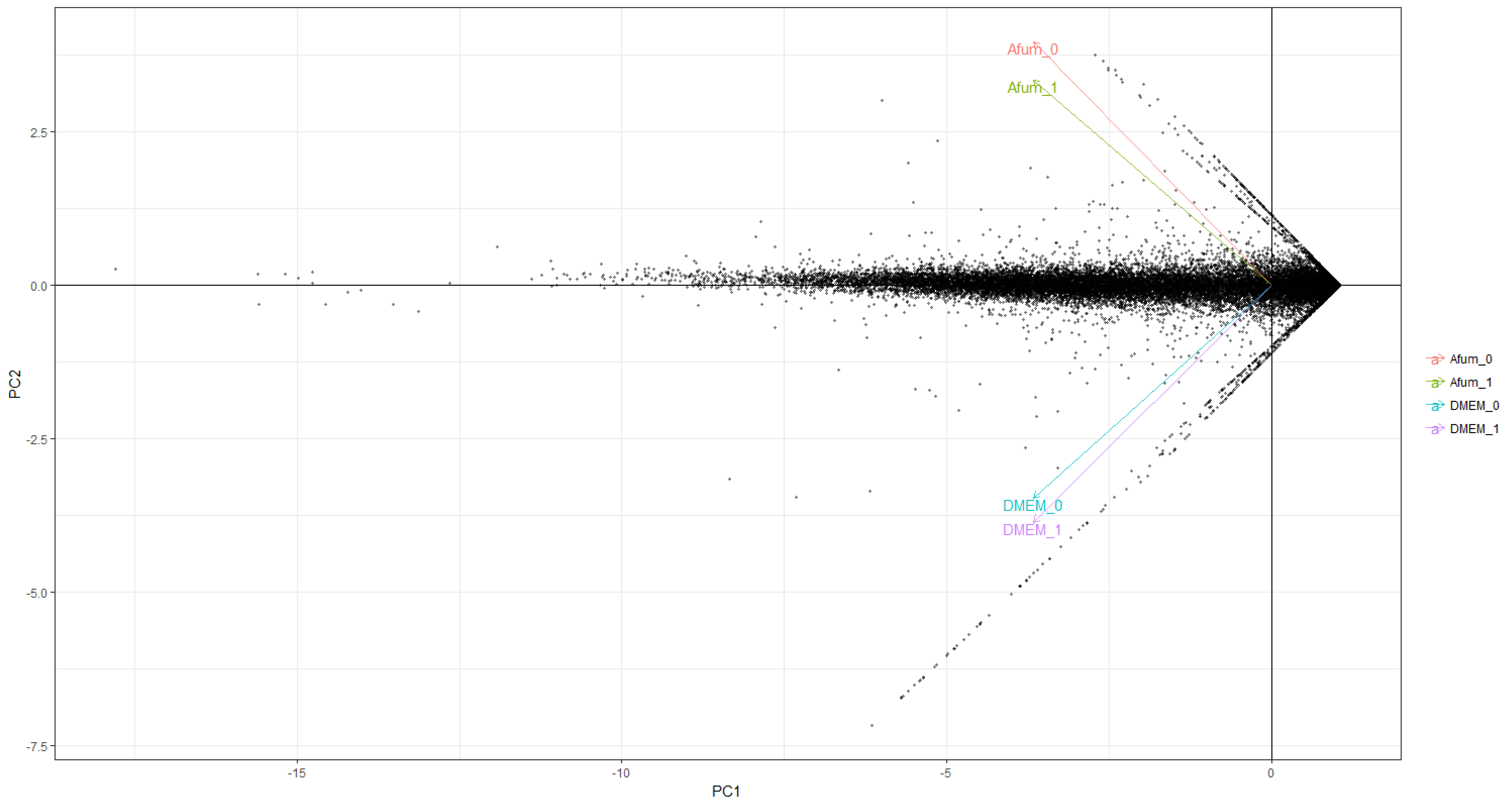

C3: Principal component analysis per sample of RNA-seq data from A549 cells in co-culture with *A. fumigatus* (Afum) and DMEM.

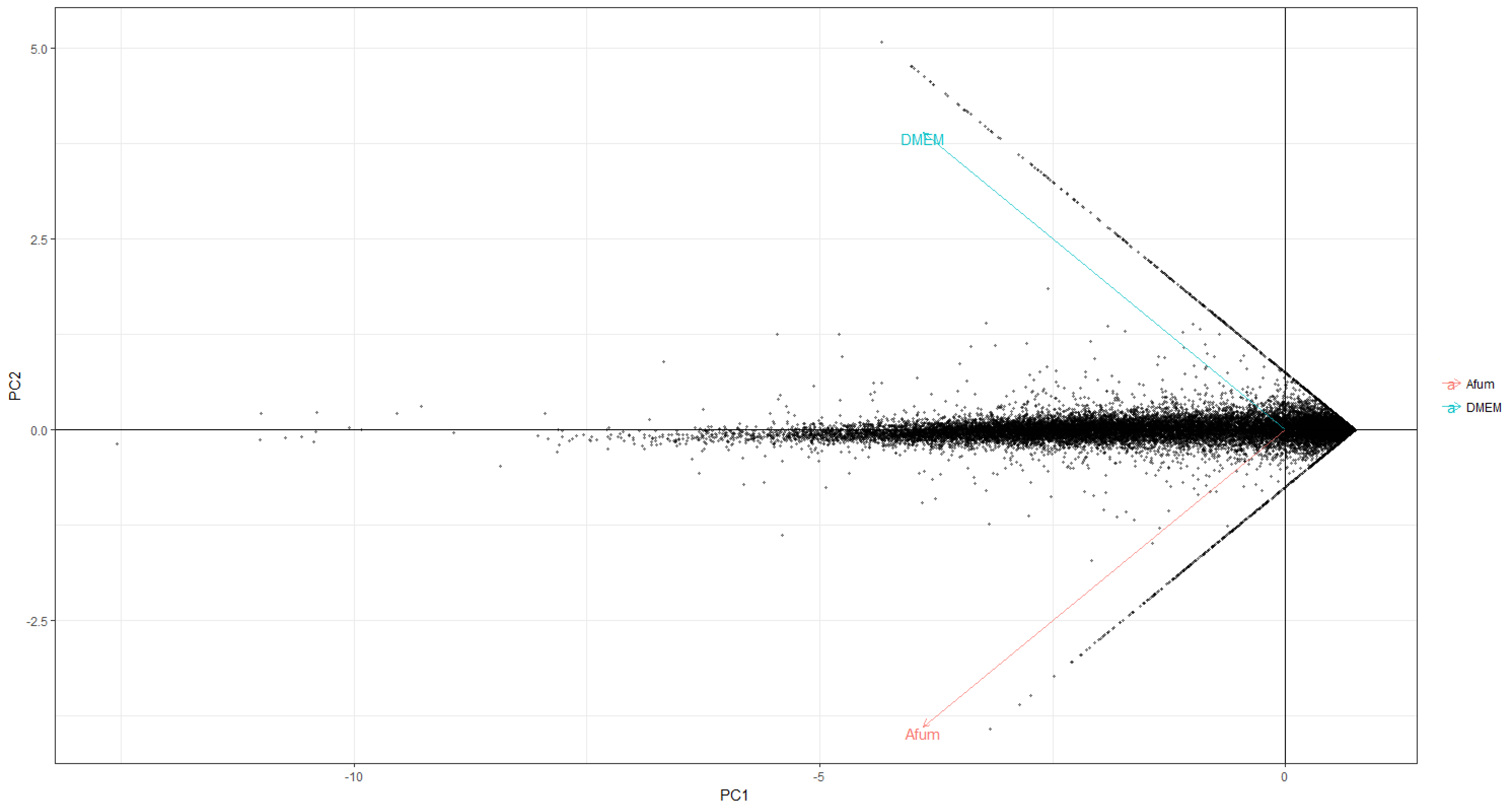

C4: Principal component analysis of RNA-seq data from A549 cells in co-culture with *A. fumigatus* (Afum) and DMEM.

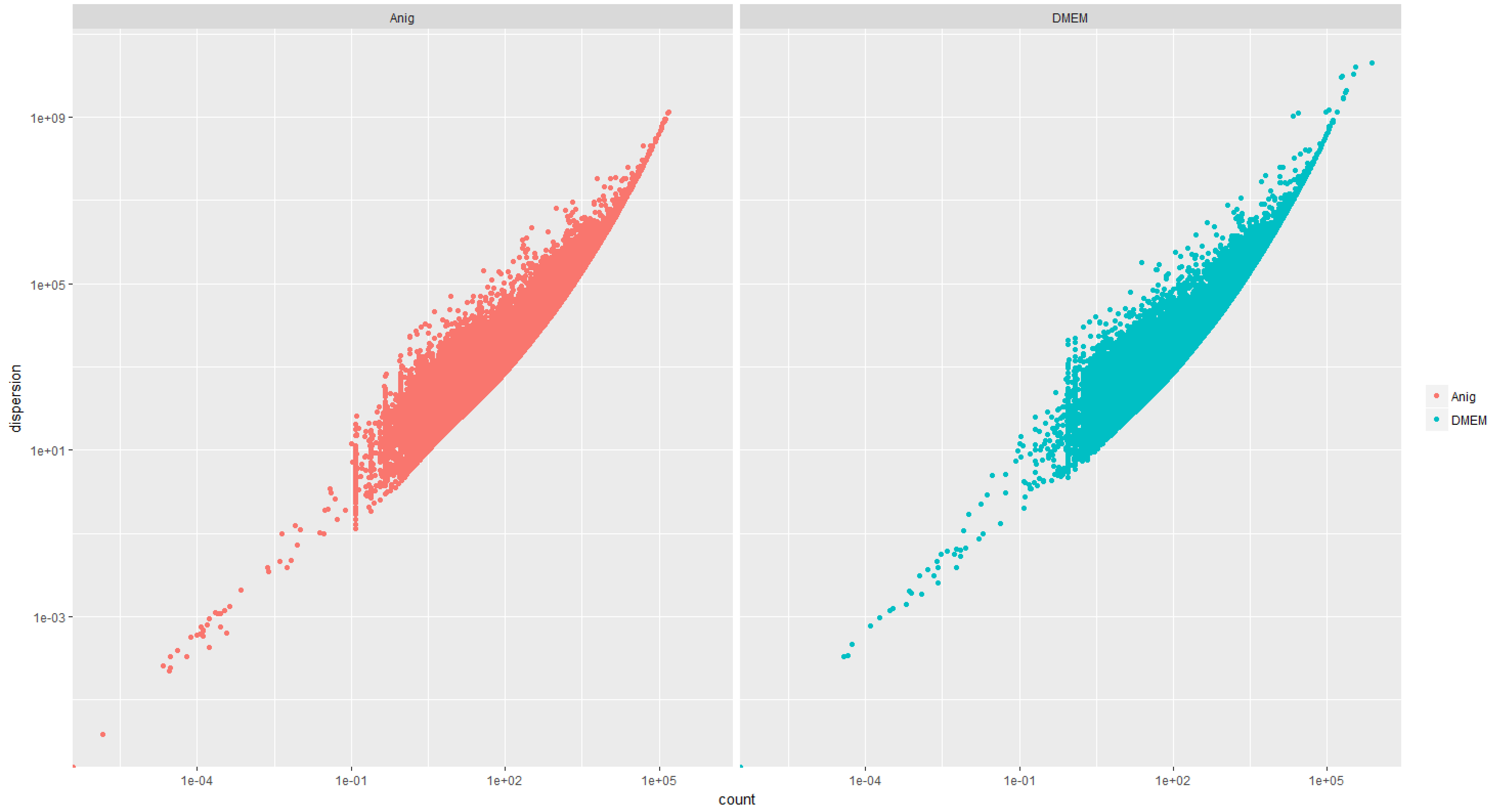

D1: Dispersion graph from A549 cells in co-culture with *A. niger* (Anig) and DMEM.

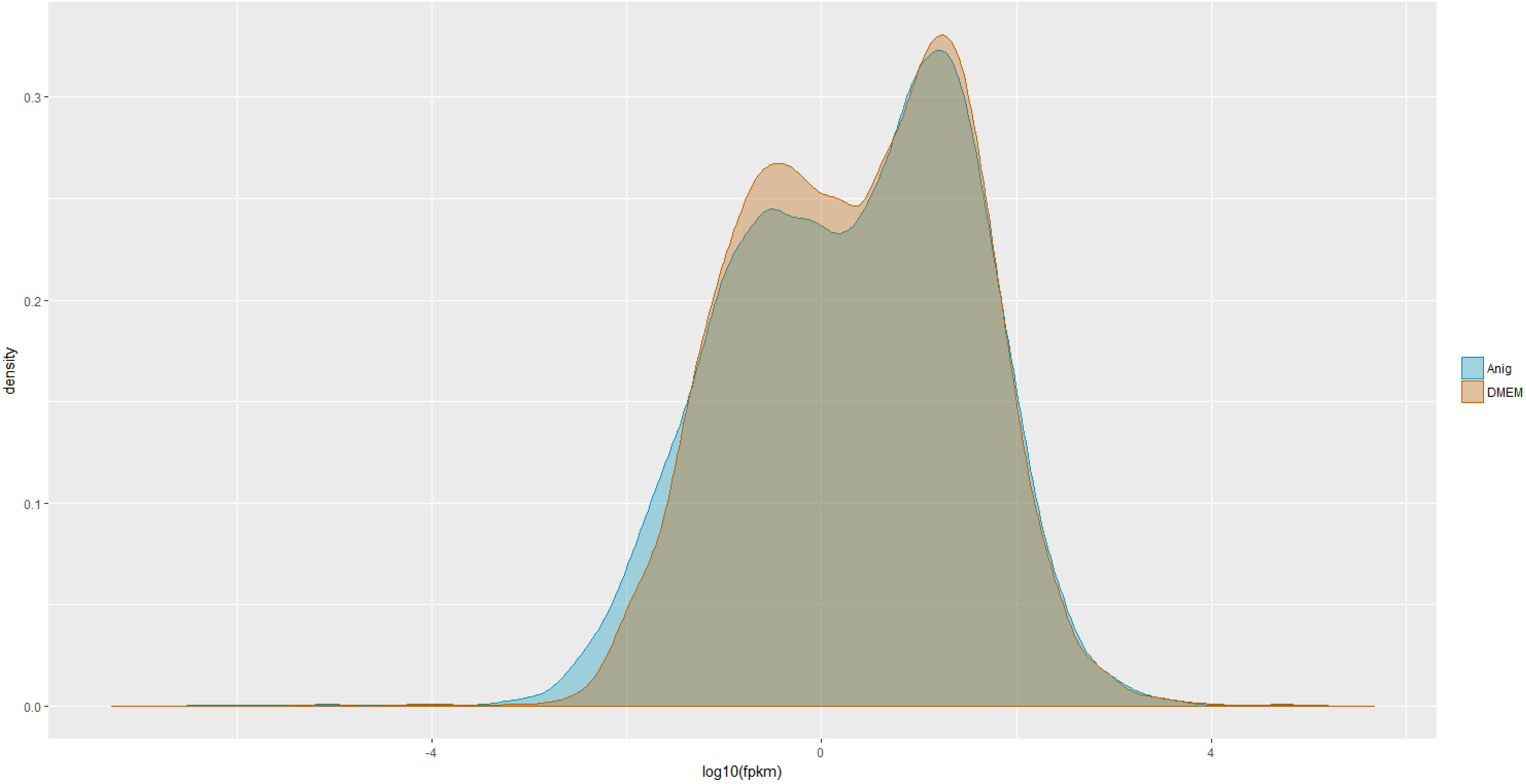

D2: Expression level distribution of mapped genes in A549 cells in co-culture with *A. niger* (Anig) and DMEM. FPKM= fragments per kilobase of transcript per million fragments mapped.

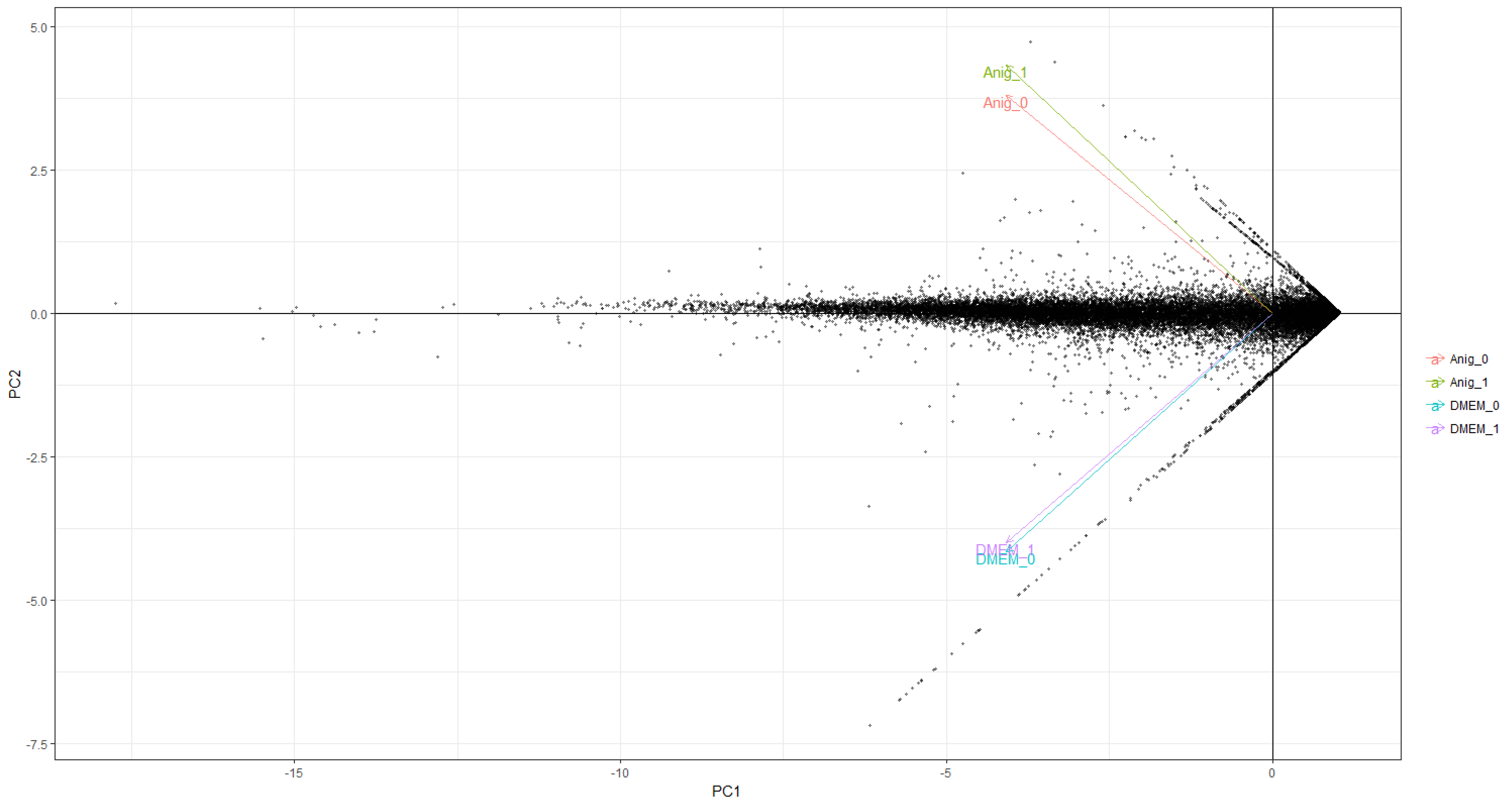

D3: Principal component analysis per sample of RNA-seq data from A549 cells in co-culture with *A. niger* (Anig) and DMEM.

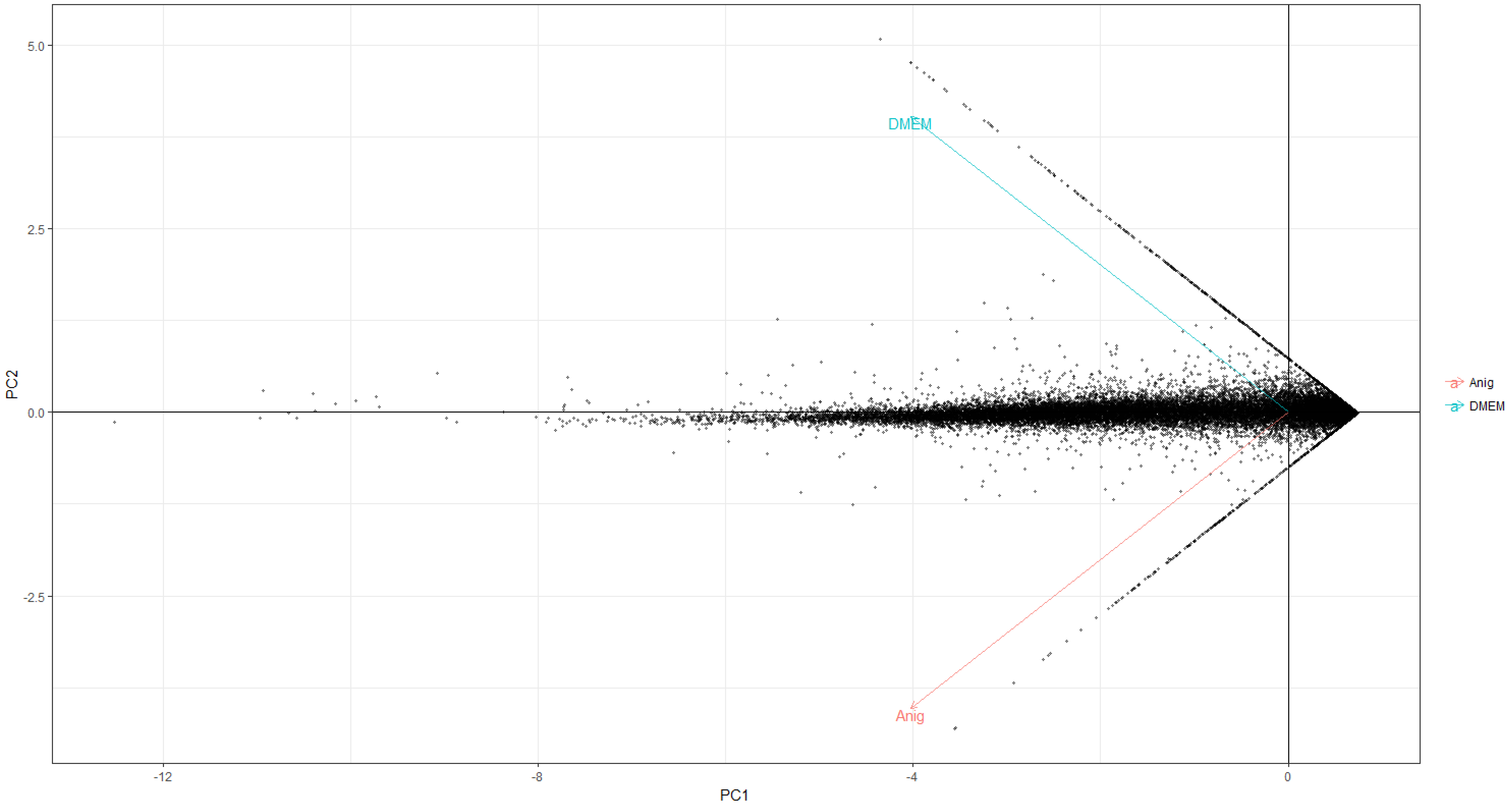

D4: Principal component analysis of RNA-seq data from A549 cells in co-culture with *A. niger* (Anig) and DMEM.
