# Supplementary material for: Expression profile analysis reveals that Aspergillus fumigatus but not Aspergillus niger makes type II epithelial lung cells less immunological alert
Source: BMC Genomics. 2018 Jul 13;19:534. doi: 10.1186/s12864-018-4895-3 (PMC6044037; doi:10.1186/s12864-018-4895-3)
Supplement: Supplementary file 7 — Table S6. Gene expression analysis using RT-qPCR. Electronic format. (DOCX 15 kb) [file 12864_2018_4895_MOESM7_ESM.docx]

**Supplementary table 6 Gene expression analysis using RT-qPCR.**

**A: IL8 expression in A549 cells after 12 hours of exposure to Tween 20 or Tween 80.**

|  | **Concentration** | **∆∆Ct** | **P Value** |  |
| --- | --- | --- | --- | --- |
| Tween 20 | 0.05% | 3.176 | 0.025 | UP |
|  | 0.005% | 1.169 | 0.654 |  |
|  | 0.0005% | 0.765 | 0.516 |  |
| Tween 80 | 0.05% | 2.474 | 0.053 |  |
|  | 0.005% | 1.815 | 0.347 |  |
|  | 0.0005% | 0.39 | 0.885 |  |

**B: 8 hour co-cultivation of A549 with *A. fumigatus* or *A. niger* spores isolated with saline in absence or presence of 0.1% Tween 20.**

|  | **Isolation spores** | **Gene** | **∆∆Ct** | **P value** |  |
| --- | --- | --- | --- | --- | --- |
| A549 + *A. fumigatus* | No Tween | IL8 | 0.524 | 0.178 |  |
|  |  | EGR1 | 2.715 | 0.006 | UP |
| A549 + *A. fumigatus* | 0.1% Tween 20 | IL8 | 0.414 | 0.145 |  |
|  |  | EGR1 | 9.528 | 0.008 | UP |
| A549 + *A. niger* | No Tween | IL8 | 1.221 | 0.685 |  |
|  |  | TNFAIP6 | 1.841 | 0.002 | UP |
|  |  | FSIP1 | 2.894 | 0.013 | UP |
| A549 + *A. niger* | 0.1% Tween 20 | IL8 | 1.205 | 0.620 |  |
|  |  | TNFAIP6 | 1.375 | 0.238 |  |
|  |  | FSIP1 | 1.484 | 0.224 |  |

**C: 12 hour co-cultivation of A549 with *A. fumigatus* or *A. niger* spores isolated with saline in absence or presence of 0.1% Tween 20.**

|  | **Isolation spores** | **Gene** | **∆∆Ct** | **P value** |  |
| --- | --- | --- | --- | --- | --- |
| A549 + *A. fumigatus* | No Tween | IL8 | 4.728 | 0.011 | UP |
|  |  | EGR1 | 59.780 | 0.002 | UP |
| A549 + *A. fumigatus* | 0.1% Tween 20 | IL8 | 3.721 | 0.011 | UP |
|  |  | EGR1 | 21.439 | 0.009 | UP |
| A549 + *A. niger* | No Tween | IL8 | 3.325 | 0.029 | UP |
|  |  | TNFAIP6 | 4.816 | 0.003 | UP |
|  |  | FSIP1 | 3.657 | 0.004 | UP |
| A549 + *A. niger* | 0.1% Tween 20 | IL8 | 2.543 | 0.030 | UP |
|  |  | TNFAIP6 | 4.748 | 0.000 | UP |
|  |  | FSIP1 | 4.591 | 0.000 | UP |
